# Conferring liver selectivity to a thyromimetic using a novel nanoparticle increases therapeutic efficacy in a diet-induced obesity animal model

**DOI:** 10.1093/pnasnexus/pgad252

**Published:** 2023-08-29

**Authors:** Ruiling Wu, Theeraphop Prachyathipsakul, Jiaming Zhuang, Hongxu Liu, Yanhui Han, Bin Liu, Shuai Gong, Jingyi Qiu, Siu Wong, Alexander Ribbe, Jewel Medeiros, Jayashree Bhagabati, Jingjing Gao, Peidong Wu, Ranit Dutta, Roman Herrera, Steve Faraci, Hang Xiao, S Thayumanavan

**Affiliations:** Department of Chemistry, University of Massachusetts, Amherst, MA 01003, USA; The Center for Bioactive Delivery, Institute for Applied Life Sciences, University of Massachusetts, Amherst, MA 01003, USA; Department of Chemistry, University of Massachusetts, Amherst, MA 01003, USA; The Center for Bioactive Delivery, Institute for Applied Life Sciences, University of Massachusetts, Amherst, MA 01003, USA; Department of Chemistry, University of Massachusetts, Amherst, MA 01003, USA; The Center for Bioactive Delivery, Institute for Applied Life Sciences, University of Massachusetts, Amherst, MA 01003, USA; Department of Chemistry, University of Massachusetts, Amherst, MA 01003, USA; The Center for Bioactive Delivery, Institute for Applied Life Sciences, University of Massachusetts, Amherst, MA 01003, USA; Department of Food Science, University of Massachusetts, Amherst, MA 01003, USA; Department of Chemistry, University of Massachusetts, Amherst, MA 01003, USA; The Center for Bioactive Delivery, Institute for Applied Life Sciences, University of Massachusetts, Amherst, MA 01003, USA; Department of Chemistry, University of Massachusetts, Amherst, MA 01003, USA; The Center for Bioactive Delivery, Institute for Applied Life Sciences, University of Massachusetts, Amherst, MA 01003, USA; The Center for Bioactive Delivery, Institute for Applied Life Sciences, University of Massachusetts, Amherst, MA 01003, USA; Department of Biomedical Engineering, University of Massachusetts, Amherst, MA 01003, USA; Molecular and Cellular Biology Program, University of Massachusetts, Amherst, MA 01003, USA; Department of Polymer Science and Engineering, University of Massachusetts, Amherst, MA 01003, USA; Department of Chemistry, University of Massachusetts, Amherst, MA 01003, USA; The Center for Bioactive Delivery, Institute for Applied Life Sciences, University of Massachusetts, Amherst, MA 01003, USA; Department of Chemistry, University of Massachusetts, Amherst, MA 01003, USA; The Center for Bioactive Delivery, Institute for Applied Life Sciences, University of Massachusetts, Amherst, MA 01003, USA; Department of Chemistry, University of Massachusetts, Amherst, MA 01003, USA; The Center for Bioactive Delivery, Institute for Applied Life Sciences, University of Massachusetts, Amherst, MA 01003, USA; Department of Chemistry, University of Massachusetts, Amherst, MA 01003, USA; The Center for Bioactive Delivery, Institute for Applied Life Sciences, University of Massachusetts, Amherst, MA 01003, USA; Department of Chemistry, University of Massachusetts, Amherst, MA 01003, USA; The Center for Bioactive Delivery, Institute for Applied Life Sciences, University of Massachusetts, Amherst, MA 01003, USA; Cyta Therapeutics, Inc., Lowell, MA 01852, USA; Cyta Therapeutics, Inc., Lowell, MA 01852, USA; The Center for Bioactive Delivery, Institute for Applied Life Sciences, University of Massachusetts, Amherst, MA 01003, USA; Department of Food Science, University of Massachusetts, Amherst, MA 01003, USA; Molecular and Cellular Biology Program, University of Massachusetts, Amherst, MA 01003, USA; Department of Chemistry, University of Massachusetts, Amherst, MA 01003, USA; The Center for Bioactive Delivery, Institute for Applied Life Sciences, University of Massachusetts, Amherst, MA 01003, USA; Department of Biomedical Engineering, University of Massachusetts, Amherst, MA 01003, USA; Molecular and Cellular Biology Program, University of Massachusetts, Amherst, MA 01003, USA

**Keywords:** liver targeting, anionic nanogel, thyroid hormone therapy, metabolic regulation, liver disease

## Abstract

Optimization of metabolic regulation is a promising solution for many pathologies, including obesity, dyslipidemia, type 2 diabetes, and inflammatory liver disease. Synthetic thyroid hormone mimics–based regulation of metabolic balance in the liver showed promise but was hampered by the low biocompatibility and harmful effects on the extrahepatic axis. In this work, we show that specifically directing the thyromimetic to the liver utilizing a nanogel-based carrier substantially increased therapeutic efficacy in a diet-induced obesity mouse model, evidenced by the near-complete reversal of body weight gain, liver weight and inflammation, and cholesterol levels with no alteration in the thyroxine (T4) / thyroid stimulating hormone (TSH) axis. Mechanistically, the drug acts by binding to thyroid hormone receptor β (TRβ), a ligand-inducible transcription factor that interacts with thyroid hormone response elements and modulates target gene expression. The reverse cholesterol transport (RCT) pathway is specifically implicated in the observed therapeutic effect. Overall, the study demonstrates a unique approach to restoring metabolic regulation impacting obesity and related metabolic dysfunctions.

**Significance Statement**

In this work, a novel nanocarrier functionalized with anionic moieties, anionic nanogels (ANGs), was developed to maximize the therapeutic benefits of thyromimetic drugs. The thyromimetic-encapsulated ANG strategy exhibits superior efficacy in restoring metabolic regulation and unravels the underlying mechanism of inhibition on liver steatosis, inflammatory injury, and fibrosis. The ANG system opens up the possibility for nanoparticle-mediated pharmaceutical strategies for related diseases such as nonalcoholic steatohepatitis (NASH), type 2 diabetes, and hyperlipidemia.

## Introduction

Disruption of metabolism regulation leads to several debilitating diseases such as obesity, inflammatory liver steatosis such as nonalcoholic steatohepatitis (NASH), type 2 diabetes, and hyperlipidemia (1–4). Among the mechanisms implicated in the regulation of metabolic balance, arguably the best characterized regulatory pathway involves the one controlled by the thyroid hormone (TH) (5–8). TH elicits its effects by binding to the TH receptors, TRα and TRβ. Effects of TH on heart, such as heart rate and rhythm, are mediated through TRα activation. On the other hand, most of the hormone-based activities in the liver (e.g. lipid-lowering) are mediated through TRβ activation (9, 10). In fact, preclinical data using TRβ selective compounds show promising results in regulating lipogenesis and metabolism (e.g. steatosis) (11–13). Despite this promise, surprisingly, the full spectrum of TH response in metabolic regulation has not been observed such as robust weight reduction in obese preclinical animal models. Indeed, small molecule thyromimetics are able to show lipid metabolism benefits, but poor TH-receptor selectivity and bioavailability have caused extrahepatic toxic effects and limited efficacy (2, 10). Thus, it is clear that selective stimulation of TRβ is a promising approach in addressing many critical gaps in metabolic disorders.

We hypothesize that a full repertoire of TH metabolic effects can be achieved by selectively delivering thyromimetic drugs to the liver as it concurrently confers TRβ targeting. This strategy offers two key advantages: (i) limited systemic exposure avoids disruption of the TH regulatory axis, as adventitious activation of TRα would lead to cardiovascular consequences such as tachycardia; (ii) increased local concentration of pharmacologically active agonist on the target receptor should confer prolonged activation of the receptor-mediated pathway regulation. In this work, we show that our nanogel-based carrier that is designed to selectively target the hepatocytic cells dramatically enhances the efficacy of a thyromimetic drug.

Anionic nanogels (ANGs), which are nanogels functionalized with anionic moieties on their surface, are especially suited for the targeted delivery of thyromimetics. As organic anion-transporting polypeptides (OATPs) are overexpressed in hepatocytes (14, 15), we hypothesized that ANGs would selectively and safely target the liver and improve the efficacy of thyromimetics. It is known that nanoparticles in the range of 10–50 nm accumulate in the liver, which acts as a major reservoir and clearance organ (16). A common fate of these nanoparticles however is that these are taken up by Kupffer cells in the liver and not hepatocytes (17). We posit that while the nanoscopic nature of ANGs would direct them to the liver, their anionic surface functionalities would direct them to the hepatocytes due to overexpression of OATP receptors. Among the surface functionalization possibilities, cationic functionalities on the surface assist in fast cellular uptake but are often quite cytotoxic (18). Charge-neutral polyethylene glycol (PEG) corona is often used in nanoparticles as it reduces recognition by macrophages and evades interaction with serum proteins, leading to prolonged circulation time (19). Therefore, charge-neutral PEG-based nanogels are used as the control neutral nanogel (NNG), as they offer to test if the OATP recognition is the selectivity driver in this system. In addition to the hepatocytic selectivity, note that the ANGs are based on cross-linked structures that stably encapsulate the drug until they are triggered in releasing the cargo by the reducing environment of the cytosol (20). This feature should ensure that the drug is not prematurely released prior to reaching the target cells. Overall, the design of liver-targeting ANGs provides a capability to reduce systemic exposure of the drug while simultaneously increasing exposure in the hepatocyte, thus potentially allowing increased safety and therapeutic efficacy.

Employing a murine diet-induced obesity-NASH (DIO-NASH) model, we demonstrate that the thyromimetic-encapsulated ANG strategy exhibits superior efficacy over the thyromimetic-alone treatment with little to no side effects against a variety of metabolic parameters. Our understanding of pathophysiology and regulation mechanism of thyromimetics in metabolic diseases, including obesity, NASH, and type 2 diabetes, is still incomplete. Herein, we analyze the effect of thyromimetic-encapsulated ANG treatment and the effect of this treatment on body weight gain, liver steatosis, inflammation injury, and fibrosis. Our data show that activation of TRβ by thyromimetics reduces de novo lipogenesis and increases hepatic cholesterol metabolism and excretion through reverse cholesterol transport (RCT) pathway. Significant body weight loss is observed as is reduction in steatosis, leading to a reduction in liver inflammation and fibrosis. More broadly, given the potential advantages associated with thyromimetics in the treatment of metabolic diseases, our ANG platform offers a robust liver delivery system for this important class of therapeutic agents.

## Results

### Construction and characterization of liver-targeting ANG system

ANGs were prepared from a random copolymer based on PEG methacrylate as the hydrophilic monomer and the pyridyl disulfide (PDS) methacrylate as the hydrophobic monomer (20). This amphiphilic polymer forms nanoassemblies in aqueous media, which was subjected to a dithiothreitol (DTT)-initiated self-cross-linking reaction in the presence of the drug cargo to generate a polymer nanogel. This was then converted to the corresponding drug-encapsulating ANG by further functionalization with 3-mercaptopropionic acid (Scheme S1). A successful synthesis of the copolymer was verified by ^1^H-NMR and gel permeation chromatography (GPC) analyses, while the cross-link density and anionic functionalization of the nanogel were confirmed by characteristic ultraviolet (UV) absorption of pyridothione byproduct at 343 nm (Fig. S1).

**Fig. 1.**
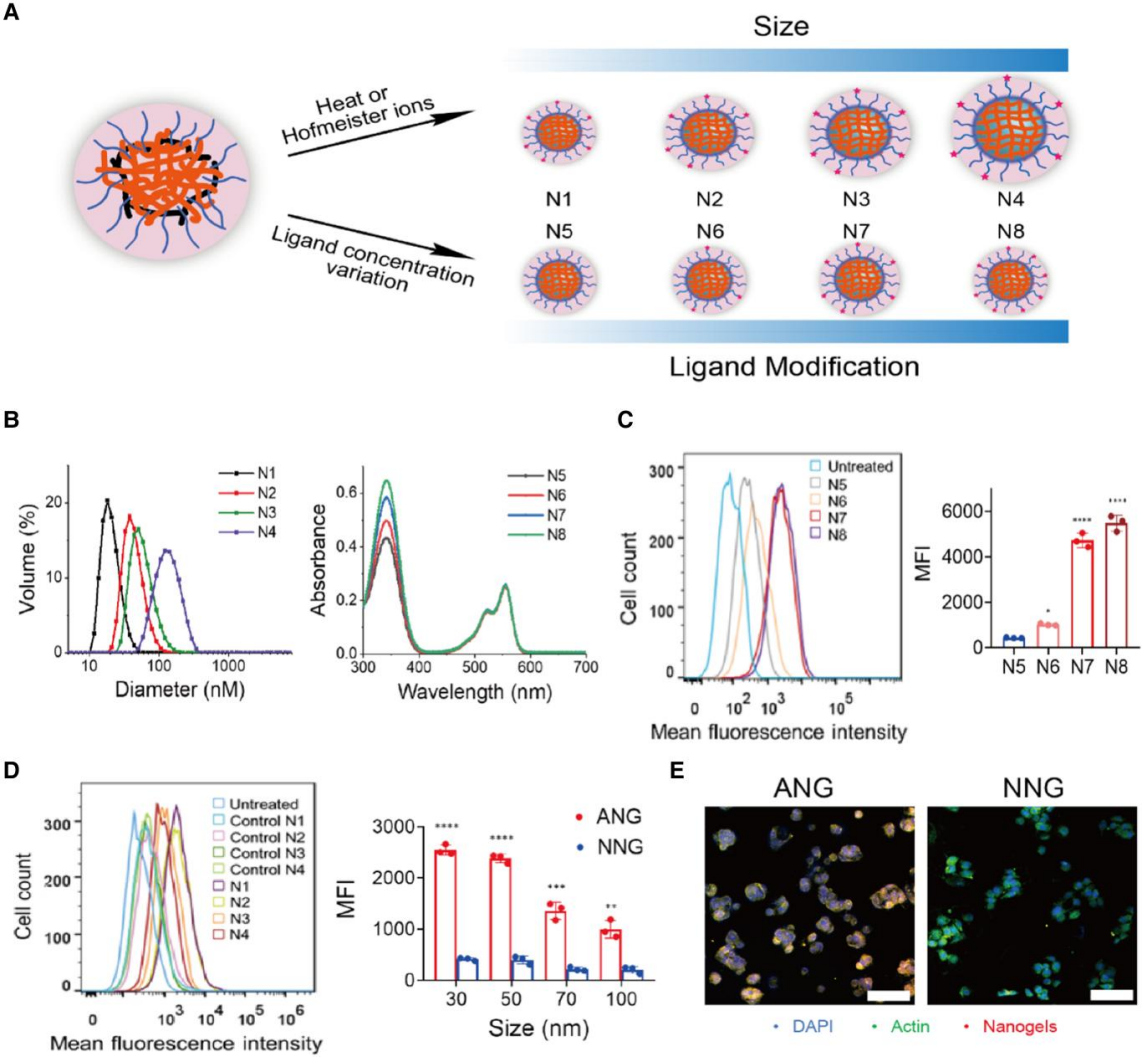
Preparation of ANG system and profile of specific liver targeting ability in vitro. A) Schematic illustration of the generation of ANG library with tunable particle size and ligand density. ANG with different sizes (N1, N2, N3, and N4) contained the same ligand modification degree (28%). ANG with different ligand modifications (N5, N6, N7, and N8) contained the same size (30 nm). B) Size and ligand modification variations of ANG library confirmed by DLS and UV. C, D) In vitro binding and uptake of the nanogels with different ligand modifications (C) and size (D) to HepG2 cells measured by flow cytometry (left) and corresponding quantification (right). Significant difference between nanogels with different physiochemical parameters was observed. Data are shown as the mean ± SD of *n* = 3 biologically independent samples. Statistical significance was calculated via two-tailed Student's *t*-test. *P < 0.05; **P < 0.01; ***P < 0.001; and ****P < 0.0001. E) Representative confocal laser scanning microscope images of Cy3–NG (N1, 30 nm, 28% ligand modification) colocalized with actin cytoskeleton (blue, nucleus; red, Cy3-NG; green, actin; scale bar: 100 µm).

As physicochemical parameters such as size and surface charge have significant biological implications in the cellular uptake and biological processes of nanoparticles (21–23), we systematically varied the particle size and anionic surface ligand density (Fig. 1A) and evaluated their effects on cellular uptake. The lower critical solution temperature behavior of these amphiphilic polymers and their differential aggregations based on Hofmeister effect provided a robust way to customize our nanogels to conveniently vary the nanogel size (24), which was characterized using dynamic light scattering (DLS) and transmission electron microscopy (TEM) (Figs. 1B and S2). These ANGs were then tested to assess cellular uptake using the well-characterized HepG2 hepatocyte cell line (25). We monitored the binding and uptake of ANGs using flow cytometry by fluorescently labeling them with Cy3 dye (Scheme S1). The effect of anionic surface functionalization was evaluated by comparing the uptake against NNGs, which only contain PEG-based surface functionalities. We found that ANGs exhibit higher levels of uptake compared with NNG, which is attributed to the higher affinity of negatively charged nanogels to OATP cell surface receptors. This assumption is supported by the fact that the degree of cellular uptake increases systematically with the surface charge density and saturates at ∼28% (Fig. 1C). Similarly, evaluation of nanogel size variations showed that 30- to 50-nm nanogels exhibit optimal cellular uptake (Fig. 1D), which is consistent with previous studies suggesting that nanoparticles that range in size between 30 and 50 nm interact more efficiently with cell membrane receptors and are subsequently internalized via receptor-mediated endocytosis (26).

Note that our goal is to deliver the thyromimetic drug into the cytoplasm of hepatocytic cells in the liver. Therefore, we were interested in identifying whether ANGs ultimately localize in the cytoplasm. Confocal microscopy was used to qualitatively determine the location of the nanogels (Figs. 1E and S4). Analysis of cells incubated with ANG showed the colocalization of fluorescent signals from Cy3-labeled ANGs and actin cytoskeleton, which is a multifunctional protein that forms microfilaments in the cytoplasm and plays an essential role in receptor-mediated endocytosis in mammalian cells (27). The distribution of nanogels in cytoplasm leads to the release of the thyromimetic cargo since the cytoplasm has a much higher glutathione (GSH) concentration, which degrades the polymer. The released thyromimetic is able to access the nucleus and interact with the TRβ1 receptor. On the contrary, NNG shows a much lower signal than the ANG and is primarily retained on the cell surface.

**Fig. 2.**
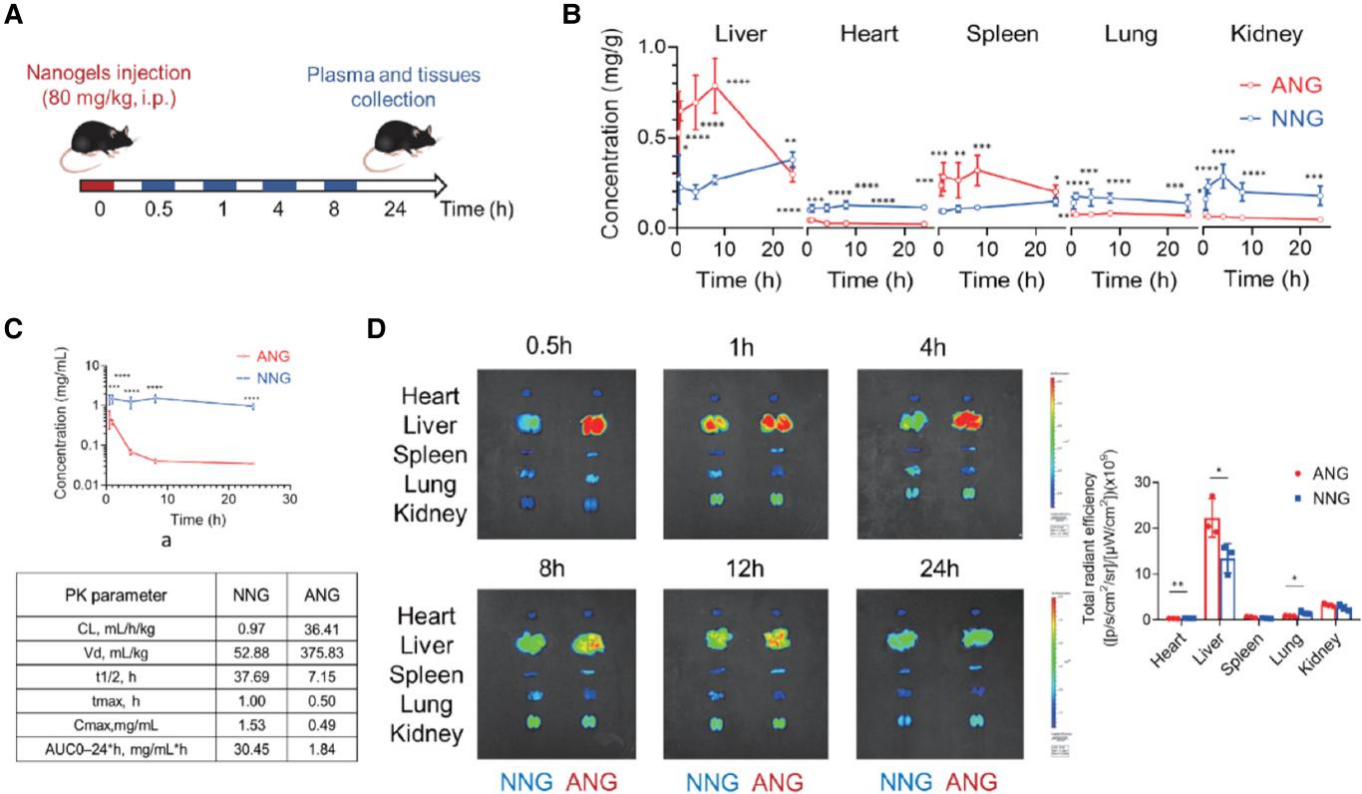
Liver-targeting ability of ANG system in vivo. A) Schematic illustration of the treatment protocol for pharmacokinetics study of ANG compared with NNG in CD-1 mice (over 0.5, 1, 4, 8, 24 h) or C57BL/6J mice (over 0.5, 1, 4, 8, 12, 24 h). i.p., intraperitoneal injection. B) Pharmacokinetics of nanogels in major mouse organs at different time points postinjection. C) Plasma pharmacokinetic curves and parameters for nanogels. AUC, area under the curve; CL, clearance; *t*_1/2_, time of half-life; Vd, volume of distribution. All data are shown as mean ± SEM (*n* = 6 biologically independent mice per group). Statistical significance was calculated via ordinary one-way ANOVA with Tukey's multiple comparison test. *P < 0.05; **P < 0.01; ***P < 0.001; and ****P < 0.0001. D) Representative ex vivo IVIS imaging of major organs from C57BL/6J mice receiving nanogels over time postinjection (left) and quantitative fluorescence intensity at 4-h time point (right). Data are shown as the mean ± SD of *n* = 3 biologically independent mice per time point. Scale bar: red to blue, signal intensity high to low. Statistical significance was calculated via two-tailed Student's *t*-test. *P < 0.05; **P < 0.01; ***P < 0.001; and ****P< 0.0001.

Next, we turned our attention to assess if the rapid uptake by the HepG2 cells translates to liver targeting by evaluating the biodistribution of ANGs (30-nm size and 28% anionic ligand density) in CD-1 mice. Organs and blood were collected and assessed at different time points postintraperitoneal injection of ANG and NNG (Fig. 2A). Indeed, distribution of the ANG in the liver was significantly higher than NNG while much lower in other organs. This difference was sustained for at least 12 h, emphasizing the liver-targeting ability of the ANG (Fig. 2B). The blood pharmacokinetic profiles of ANG and NNG were also examined (Fig. 2C), which showed that ANG was cleared rapidly with a larger distribution volume and a shorter plasma half-life. In contrast, NNG exhibited an increased retention performance in the circulatory system with low distribution volume. It is reasonable to suggest that the rapid clearance of ANG resulted from the liver-homing capability due to interaction with the OATP receptors and the resultant uptake into the hepatocyte. To test if liver accumulation is a strict function of mouse genetics, C57BL/6J mice were treated with the nanogels using identical conditions as with CD-1 mice (Fig. 2A). The organs were collected and fluorescently imaged at different time points; the results show similar biodistribution profiles (Figs. 2D and S5). Therefore, the ANG system shows excellent liver targeting ability, justifying subsequent thyromimetic-ANG formulation and testing in the DIO mouse model.

### Evaluation of potential therapeutic effect of CGS-ANG

**Fig. 3.**
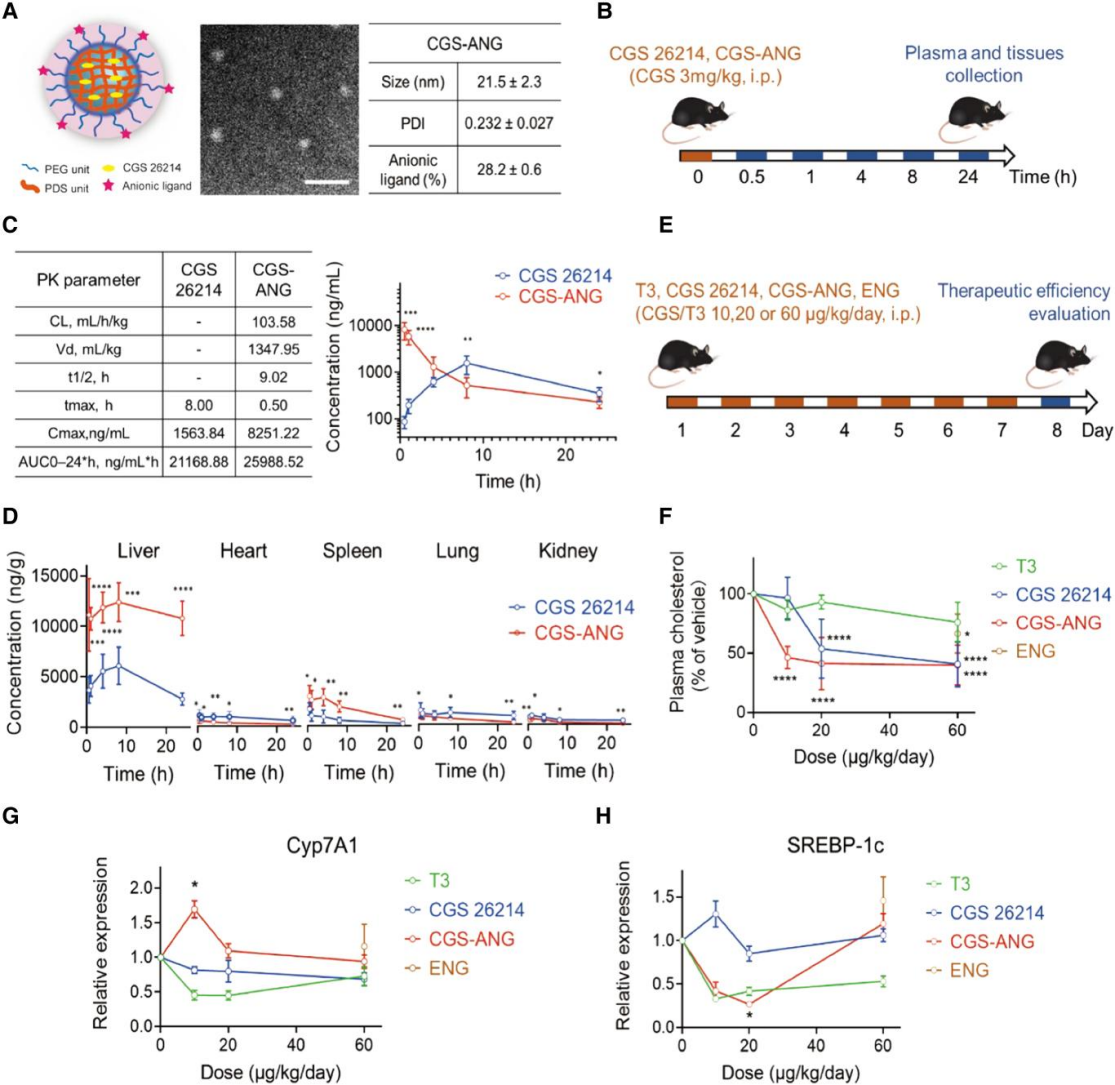
Delivery efficiency of CGS-ANG to mice liver and potent therapeutic activity. A) Representative images and characterization of CGS-ANG. Data was shown as the mean ± SD from three experiments. PDI, polydispersity index. Scale bar of TEM images of CGS-ANG: 250 nm. B) Schematic illustration of the treatment protocol for pharmacokinetics study of CGS-ANG in C57BL/6J mice. i.p., intraperitoneal injection. C) Plasma pharmacokinetic parameters (left) and curve (right) for free-form CGS 26214 and CGS-ANG. AUC, area under the curve; CL, clearance; *t*_1/2_, time of half-life; Vd, volume of distribution. D) Pharmacokinetics of free-form CGS 26214 and CGS-ANG in major mouse organs at different time points postinjection. *P < 0.05; **P < 0.01; ***P < 0.001; and ****P < 0.0001. E) Schematic illustration of the treatment protocol of evaluation study for potent therapeutic effect of CGS-ANG in normal C57BL/6J mice. CGS-ANG loaded with CGS 26214 at the dose that is the same as corresponding CGS 26214 groups. ENG is only shown as one spot at the highest dose of ANG, equivalent to ANG used in the highest dose CGS-ANG group (60 μg/kg/day). F) Effects of increasing dose of CGS-ANG, CGS 26214, T3, and ENG on total plasma cholesterol of normal mice. Results are expressed as fold change relative to the PBS vehicle-treated control. G, H) Relative mRNA expression of Cyp7A1 (G) and SREBP-1c (H) in livers from the PBS, CGS-ANG, CGS 26215, T3, and ENG treatment groups. *P < 0.05; **P < 0.01; ***P < 0.001; and ****P < 0.0001 compared with control in F)–H). All data are shown as mean ± SEM (*n* = 6 biologically independent mice per group). Statistical significance was calculated via ordinary one-way ANOVA with Tukey's multiple comparison test.

Having confirmed the liver targeting ability, we then generated thyromimetic-encapsulated ANGs. The thyromimetic agent we used here is Axitirome (also known as CGS 26214), a potent, liver-selective agonist for the TRβ receptor that has demonstrated cholesterol-lowering activity in both rat and dog models (28–30). The ANG encapsulating CGS 26214 (CGS-ANG) was assembled by incorporating the hydrophobic drug into copolymer micelles followed by DTT-based cross-linking reaction (20) (Fig. 3A and Scheme S1). The loading capacity varied with drug feeding amount and the encapsulation efficiency is typically around 50%.

To test if liver-specific localization of CGS 26214 can be achieved using the CGS-ANGs in vivo, we evaluated the pharmacokinetics and biodistribution of the nanogels in a C57BL/6J mice model (Fig. 3B). Plasma and tissues were collected and analyzed by liquid chromatography–tandem mass spectrometry (LC–MS/MS) after sacrificing the mice at predetermined time points after dosing. The pharmacokinetic profile of the CGS-ANG closely resembled the profile of the ANGs. CGS 26214 that was loaded into the ANG underwent rapid distribution to the organs. Free-form CGS 26214 reached systemic circulation much more slowly and showed low systemic exposure (Fig. 3C), which is likely due to poor absorption and distribution. In contrast, CGS-ANG showed a rapid and significantly enhanced distribution in the liver right after administration compared to the free-form CGS 26214. In the ANG-based formulation, substantially less CGS 26214 was detected in extrahepatic tissues, demonstrating that the CGS-ANG has significantly better liver-targeting capabilities, which holds the promise of reducing thyromimetic-associated systemic side effects. Also, the concentration of CGS 26214 (from the CGS-ANG) in the liver was maintained at a high level even after 24 h, suggesting that ANGs are an ideal carrier for the targeted delivery and release of CGS 26214 into the liver (Fig. 3D).

Prior to initiating studies in the DIO murine disease model, we verified the therapeutic potency of CGS-ANG using normal C57BL/6J mice (Fig. 3E). THs are known to influence metabolism with profound effects on hepatic fatty acid and cholesterol metabolism, which is potentially beneficial in the treatment of various metabolic disorders (5–8). To evaluate the effect of CGS-ANG on homeostasis of major lipid components, lipoprotein concentrations in plasma were analyzed from mice that were given seven daily doses. All treatments yielded dose-dependent reductions of plasma cholesterol and the response to CGS-ANG was stronger than TH (3,5,3′-triiodo-l-thyronine [T3]) and free-form CGS 26214 (Fig. 3F). At a dose of 10 µg/kg/day, plasma triglyceride levels were only slightly reduced by the CGS-ANGs or free-form CGS 26214. T3, however, increased plasma triglyceride at all doses (Fig. S6).

TH and the liver are intimately linked in regulating lipid metabolism at transcriptional level. Thus, the above results prompted us to assay for hepatic expression of receptors involved in related pathways, including low-density lipoprotein (LDL) clearance, RCT, and lipogenesis (2). CGS-ANG significantly induced gene expression of scavenger receptor class B member 1 (SR-B1) and cholesterol 7 alpha-hydroxylase (Cyp7A1) at the 10 µg/kg/day dose, suggesting it might influence plasma cholesterol levels via the RCT pathway. The other two treatments did not show significant effects on these gene products and even had opposite effects at some doses (Figs. 3G and S7). Although several studies show that the major mechanism for cholesterol clearance in rodents is by inducing the LDL receptor (LDLR) (31–33), we did not detect significant changes in hepatic LDLR and sterol regulatory element-binding protein-2 (SREBP2) mRNA levels (Fig. S7). Sterol regulatory element-binding protein-1c (SREBP-1c) genes that are involved in fatty acid synthesis and assembly of very-low-density lipoprotein (VLDL) particles were inhibited by CGS-ANG to about 30% of control at low dose (Fig. 3H). T3 also exerted the same function even though it is accompanied by a slightly increased plasma triglyceride level, likely because TH is an activator of de novo lipogenesis (2). In addition, empty nanogels (ENGs) did not show differential actions compared with the saline group even at the highest dose (Figs. 3, S6, and S7). Collectively, these findings demonstrate the possible pathways for the involvement of CGS 26214 in lipid metabolism and highlight the unique potential of the ANG vehicle as a delivery agent.

### Alleviation of GAN diet-induced disease by CGS-ANG

**Fig. 4.**
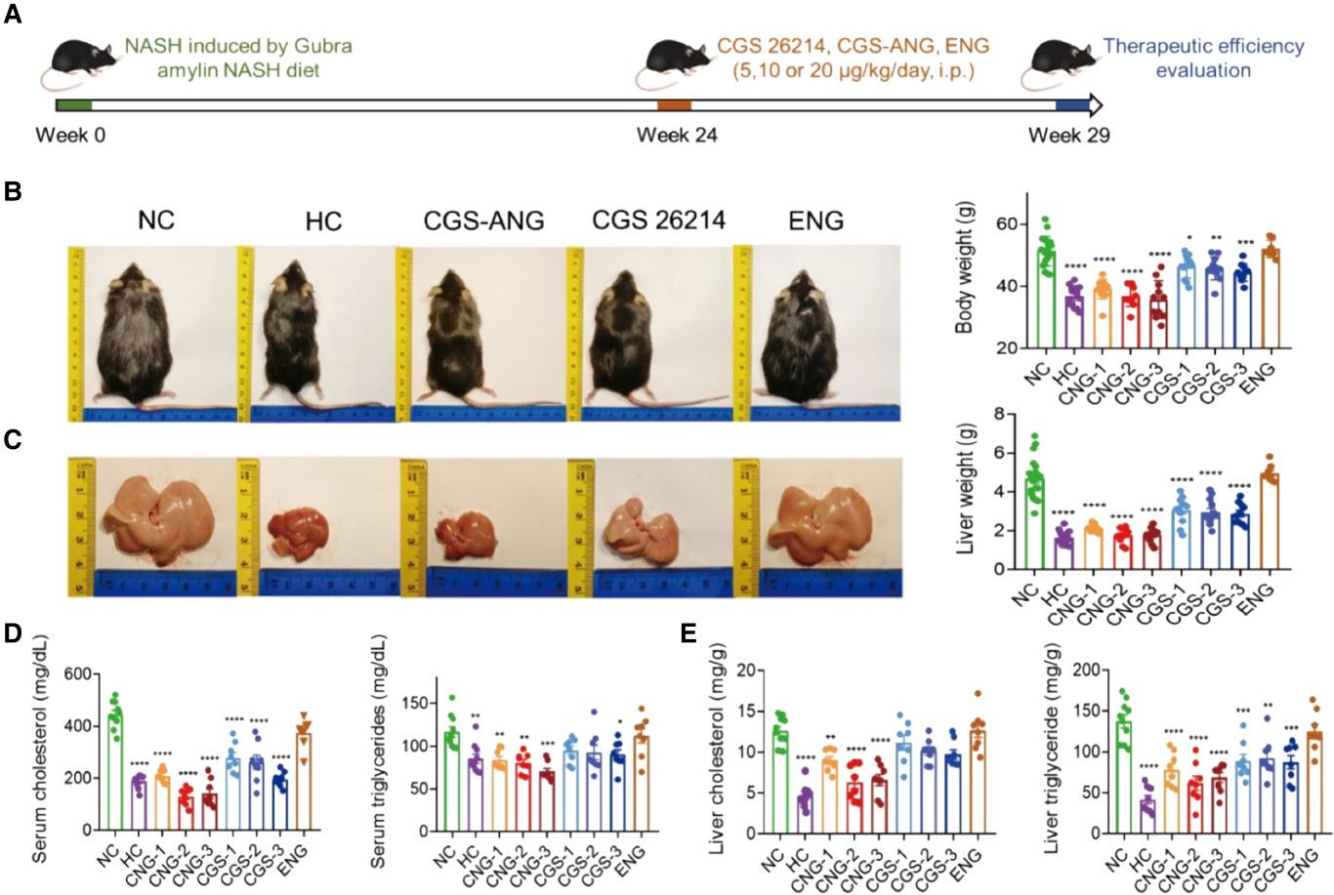
In vivo lipid-regulating effects of CGS-ANG in GAN diet-induced disease model. A) Schematic illustration of protocol for fully developed disease establishment in C57BL/6J mice and therapeutic study of CGS-ANG. DIO-induced mice treated with the vehicle were labeled as NC. Mice fed with normal chow diet and treated with the vehicle were labeled as HC. B) Representative images (left) and body weights (right) of HC mice or DIO mice treated with vehicle (NC), CGS 26214 of three doses (CGS 1, 2, and 3 and—CGS 5, 10, and 20 µg/kg/day), CGS-ANG of three doses (CNG 1, 2, and 3—CNG 5, and 10, and 20 µg/kg/day) and ENG. C) Macroscopic images of the livers (left) and liver mass (right) of mice in each group indicated. Representatives from dose 2 of CGS 26214 and CGS-ANG groups (CGS-2 and CNG-2) were shown in the pictures. *n* = 8–20 biologically independent mice per group (*n* = 15 for HC group; *n* = 20 for NC group; *n* = 12 for CGS 26214 and CGS-ANG groups; and *n* = 8 for ENG group). D, E) Effects of treatments on total serum cholesterol and triglyceride (D) and total liver cholesterol and triglyceride (E) of mice. *n* = 8–10 biologically independent mice per group (*n* = 10 for HC and NC groups; and *n* = 8 for CGS 26214, CGS-ANG, and ENG groups). All data are shown as mean ± SEM. Statistical significance was calculated via ordinary one-way ANOVA with Tukey's multiple comparison test. *P < 0.05; **P < 0.01; ***P < 0.001; and ****P < 0.0001 compared with NC control.

Next, we established a DIO disease model by maintaining C57BL/6J mice on a Gubra amylin NASH (GAN) diet, which has been shown to not only increase body weight but also reliably induce metabolic and liver histopathological changes (34). Our interest in this model emanates from the possibility of broadly capturing the impact of the thyromimetic treatment not only in obesity but also in understanding the potential impact in steatotic liver disorders, such as NASH. The effect of CGS-ANG treatments after 3-month feeding would evaluate the preventative effects (preventative study; Fig. S8), while treatment after the fully developed disease (6 months) would be regarded as the therapeutic effect (therapeutic study; Fig. 4A). After GAN diet feeding, mice were injected intraperitoneally daily by free-form CGS 26214 (CGS-1,2,3), CGS-ANG with three doses (CNG-1,2,3), and empty ANG (ENG) for 5 weeks under GAN diet. CGS-ANG loaded with CGS 26214 at the dose that is the same as the corresponding CGS 26214 groups. ENG represents empty ANG dose where the dosage of the nanogel is the same as the highest dose of the CGS-ANG group used for toxicity evaluation. In the preventative study, CGS-ANG administration gradually reduced the body weight of obese mice to the same magnitude as the healthy control (HC) mice at the end of administration. Free-form CGS-26214, however, only slowed the increase of body weight (Fig. S9). Meanwhile, food intake was similar in each treatment group of obese mice (Fig. S10). Reduction of body weight was associated with a significant decrease in liver weight in CGS-ANG treatment groups at all doses (both absolute values and relative to body weight; Table S1). This was in line with a significant decline of liver lipid content (Fig. S11). Epididymal white adipose tissue (eWAT) was also weighed (Table S1). eWAT is a highly plastic organ that orchestrates systemic lipid homeostasis with the liver. The increase in eWAT is believed to be due to excess energy accumulation in the form of lipids and proinflammatory adipokine release, which directly act on the liver to promote disease (35). Similar to what was seen with body weight reduction, CGS-ANG treatment led to a complete reversal of eWAT weight increase, indicating the superior efficacy of the encapsulated thyromimetic versus free-form thyromimetic. In addition, circulating lipid levels and plasma transaminase activities (Fig. S11) were also reduced. Heart weights were recorded to evaluate potential cardiotoxicity. No effect was observed at any dose of CGS-ANG on heart weight, while the free-form CGS 26214 (at the highest dose) caused a significant increase compared with negative control (NC) mice (Table S1). The reduction in body and tissue weights and the decrease in lipid and transaminase levels of mice under CGS-ANG administration suggest that it exerts a preventative effect on the progression of NASH.

**Fig. 5.**
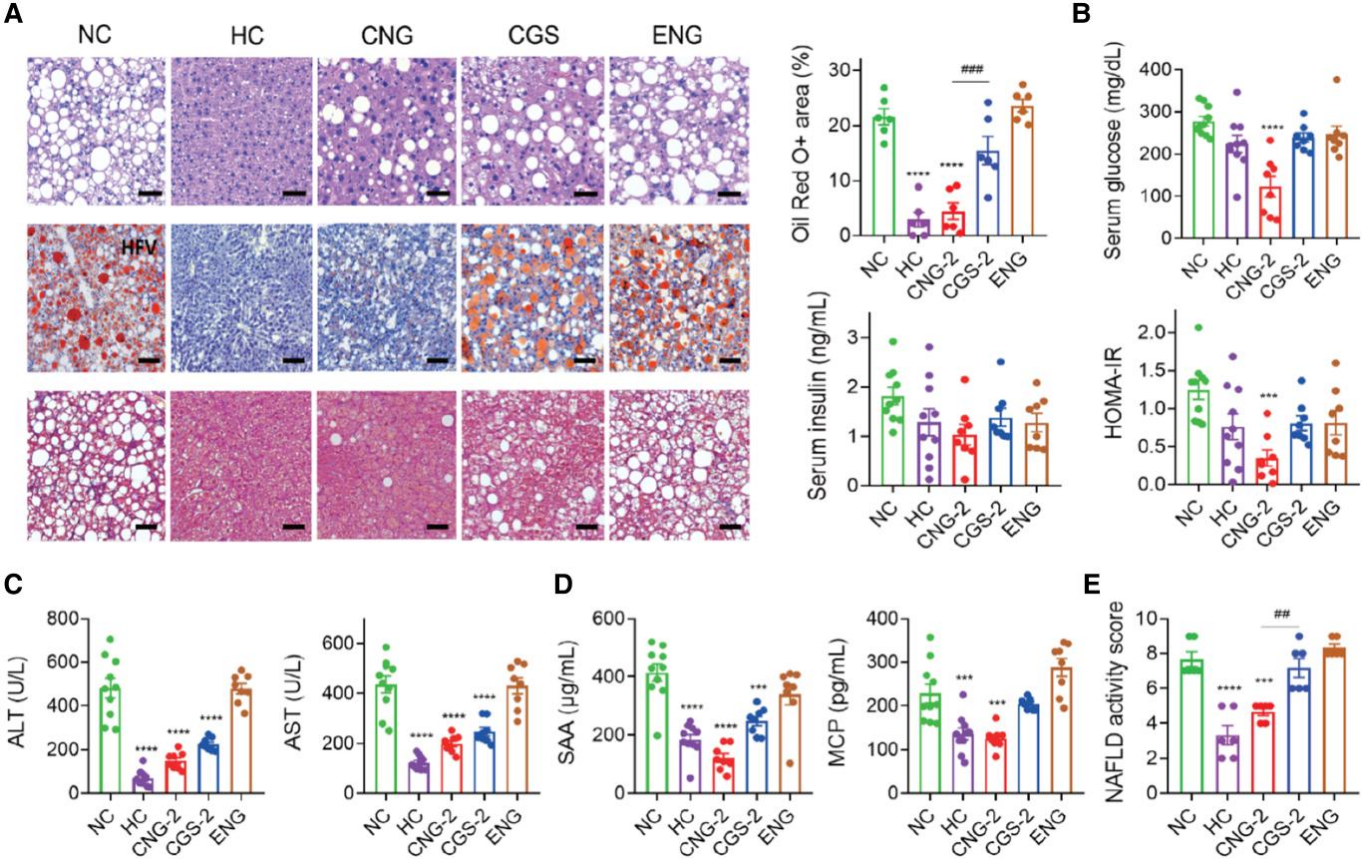
In vivo antisteatotic effect of CGS-ANG in GAN diet-induced disease model. A) Staining of liver sections for H&E (upper panels; scale bar: 50 µm), oil red O (middle panels; scale bar: 100 µm), and MT (lower panels; scale bar: 50 µm). Lipid droplet areas were quantified from three randomly chosen fields per liver section from individual mice using six mice per group. B–D) Effects of treatments on serum glucose, insulin, HOMA-IR levels (B), serum ALT, AST (C), proinflammatory cytokines SAA, and MCP (D) of mice. *n* = 8–10 biologically independent mice per group (*n* = 10 for HC and NC groups; and *n* = 8 for CGS 26214, CGS-ANG, and ENG groups). E) NASs of mice after different treatments. Cases with NAS ≥ 5 were classified as pathological. *n* = 6 biologically independent mice per group. All data are shown as mean ± SEM. Statistical significance was calculated via ordinary one-way ANOVA with Tukey's multiple comparison test. *P < 0.05; **P < 0.01; ***P < 0.001; ****P < 0.0001 compared with NC control. ^#^*P* < 0.05; ^##^*P* < 0.01; ^###^*P* < 0.001; and ^####^*P* < 0.0001 for the CNG-2 to CGS-2 comparisons.

In the therapeutic-intervention study, C57BL/6J mice were fed 24 weeks of GAN diet, after which the mice were obese and developed obvious signs of pathophysiological characteristics (Fig. 5E). The body and tissue weights of these mice were nearly normalized upon treatment with CGS-ANG, which were confirmed by gross morphologies (Fig. 4B and C and Table S1). The ability of CGS-ANG to regulate lipid homeostasis was illustrated by reduced serum and hepatic lipid levels (Fig. 4D and E). Histological examination using hematoxylin and eosin (H&E) staining and oil red O staining confirmed the antisteatotic effect of CGS-ANG, including a reduction in the overall area of lipid droplets, ballooned hepatocytes, and steatosis (Fig. 5A). Decrease of homeostasis model of assessment for insulin resistance (HOMA-IR) suggests improvement in insulin resistance, which is partially caused by hepatic steatosis (Figs. 5B and S12).

Next, biomarkers in metabolic inflammation, hepatocyte injury, and fibrosis triggered from the imbalance of lipid homeostasis were evaluated. The suppressed inflammation of DIO mice exposed to CGS-ANG was verified by reduced levels of ALT, AST (Figs. 5C and S13), serum amyloid A (SAA), and monocyte chemoattractant protein-1 (MCP-1) (Figs. 5D and S13). Masson's trichrome (MT) staining was used to detect fibrosis in liver sections. The NC group mice exhibited mild fibrosis while CGS-ANG significantly lowered the ECM depositions (Fig. 5A). The non-alcoholic fatty liver disease (NAFLD) activity score (NAS), which considers steatosis, lobular inflammation, and hepatocellular fibrosis, was assessed in H&E staining and MT staining liver sections by pathologists blinded to the treatment groups using a modified Kleiner scoring system that is applicable to all stages of NAFLD etiology and could generally be used in preclinical rodent studies (36, 37). Our results show a significant decrease in the NAS of CGS-ANG groups compared with NC mice (Figs. 5E and S14). ENG did not cause a significant difference in primary biomarkers compared with NC mice. All mice were well tolerated throughout the treatment period with no adverse side effects or signs of toxicity, suggesting ENG was well tolerated.

**Fig. 6.**
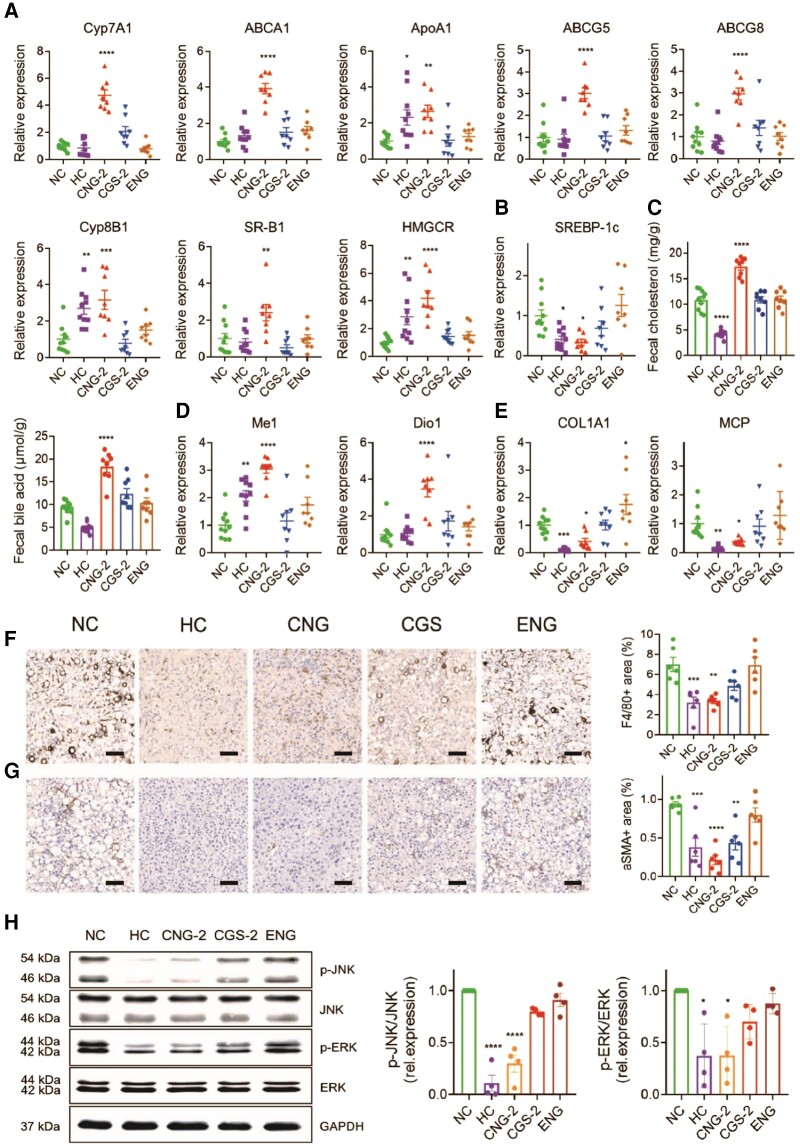
Exploration of therapeutic mechanism of CGS-ANG. A, B) Relative mRNA expression of genes related to hepatic cholesterol metabolism (A) and lipogenesis (B) in livers of mice after treatments. C) The levels of fecal cholesterol and fecal bile acid in mice after treatments. D, E) Relative mRNA expression of genes related to THR activation (D) and inflammation or fibrosis (E) in livers of mice after treatments. *n* = 8–10 biologically independent mice per group (*n* = 10 for HC and NC groups; and *n* = 8 for CGS 26214, CGS-ANG, and ENG groups) for A) and B). F, G) Representative immunohistochemistry images and quantitative analysis of liver F4/80 (F) and α-SMA (G). Scale bar: 100 µm. Positive areas were quantified from three randomly chosen fields per liver section from individual mice using six mice per group. *n* = 6 biologically independent mice per group. H) The protein levels of phospho-ERK, ERK, phospho-JNK, and JNK in the livers of different treatment groups. The band densities for phospho-ERK, ERK, phospho-JNK, and JNK were first divided by GAPDH density to correct for very small loading differences. Then, the levels of phospho-JNK/JNK and phospho-ERK/ERK were normalized to NC group. *n* = 8 biologically independent mice per group and every two mice were pooled as one sample. Cumulative densitometric analyses were performed from four independent gels. All data are shown as mean ± SEM. Statistical significance was calculated via one-way ANOVA with Dunnett's test for A), B), D), and E) comparing each group to NC control and ordinary one-way ANOVA with Tukey's multiple comparison test for C), F), G), and H). *P < 0.05; **P < 0.01; ***P < 0.001; and ****P < 0.0001 compared with NC control.

Our current study shows that encapsulation of CGS 26214 in the ANG provides additional metabolic benefits, including reversal of body weight gain with no changes in systemic T4 or TSH levels (Fig. S15). In order to investigate the molecular mechanisms underlying the observed regression, we first determined expression levels of genes involved in lipid metabolism. The results confirm our hypothesis from our evaluation study in normal mice. CGS-ANG significantly induces genes in RCT pathway (Figs. 6A and S16) while gently attenuating genes related to lipogenesis and fatty acid metabolism (Figs. 6B and S17). Induction of hepatic Cyp7A1 and ATP-binding cassette subfamily G member (ABCG5/G8) gene expressions were also mirrored by stimulated biliary secretion and fecal excretion of cholesterol and bile acids (Figs. 6C and S18). The unchanged expression of LDLR and SREBP-2 suggests little contribution from the LDLR-mediated uptake to the overall efficacy in cholesterol lowering (Fig. S19). In addition, we confirmed CGS-ANG treatments exert their functions mainly by activation of TRβ, as revealed by the strong induction of the TRβ-target genes malic enzyme 1 (ME1) and deiodinase 1 (Dio1) (Figs. 6D and S19).

CGS-ANG also causes a robust reduction in the expression of mRNAs related to hepatic inflammation and fibrosis (Figs. 6E and S20). Although the reductions in genes F4/80 and α-smooth muscle actin (α-SMA) are not statistically significant, decreases in F4/80+ macrophages and α-SMA+ cells were observed in immunohistochemistry analysis, suggesting the regulation of CGS-ANG involved posttranscriptional mechanisms (Figs. 6F and G and S21). Hepatic inflammatory cytokines have been shown to activate the mitogen-activated protein kinase (MAPK) signaling pathways, including extracellular signal-regulated kinase (ERK) and c-Jun N-terminal kinase (JNK), which are involved in the repression of Cyp7A1 expression mediated by FGF15/19 (38, 39). Thus, we explored JNK and ERK signaling under the regulation of CGS-ANG. The expression of phosphorylated ERK1/2 (p-ERK) and p-JNK are reduced in the livers of CGS-ANG–treated mice compared to the vehicle group (Figs. 6H and S22).

## Discussion

The activation of TRβ on liver is associated with systemic lipid lowering, increased bile acid synthesis, and fat oxidation. However, the positive lipid-regulating effects of TH and their mimetics are counterbalanced by their low biocompatibility and harmful effects in extrahepatic axis (9–13). With the observed data, ANGs used in this work can overcome this limitation. ANGs provide two significant advantages: (i) reduce off-target side effects that can occur with drugs that circulate systemically and (ii) potentially increase the efficacy of the encapsulated drug by attaining higher drug concentrations in the hepatocyte that would not be achieved otherwise.

The proposed polymeric nanogels are based on disulfide cross-linkers and thus are sensitive to GSH concentration. These disulfide cross-links are known to be stable in blood, where the GSH concentration is micromolar (20). However, these bonds can be cleaved inside cells due to the elevated glutathione concentrations, which are in the millimolar range (20). Thus, ANGs provide specific advantages in stably encapsulating the drug until it reaches the cellular interior, upon which it releases the drug. The innovation also lies in the invention of a system where one has a significant amount of tunability in all the critical factors desired in a nanoscopic delivery vehicle (e.g. size, encapsulation stability/release kinetics, loading capacity, and surface functional groups). The nonglassy polymeric assembly allows for significant drug loading but locks in the drug molecules due to an in situ cross-linking reaction to afford stable encapsulation.

To realize high-affinity interaction between anionic functionalities located on the exterior of the ANGs and hepatocytes, polymeric nanogels were functionalized with small molecule anionic moieties in a facile and selective process, providing a unique advantage over other systems. We would be remiss if we did not compare this to other targeted delivery approaches, such as antibody–drug conjugate (ADC) platforms. In comparing ANGs to ADC (or ligand-conjugated targeted approaches), there are two critical advantages to ANGs: (i) first is drug cargo density; a 70-nm nanogels would contain ∼10^3^ to 10^4^ drug molecules, thus providing a significant increase in drug molecules per targeting antibody or ligand (compared with the single digit ratio found in conventional ADCs) (40–43). This increase in drug/antibody ratio is a differentiating factor between the targeted ANGs and ADCs, as it provides an ability to modulate drug level in hepatocytes to arrive at the appropriate drug concentration. (ii) Second is complex chemistry; since the drug is noncovalently encapsulated in the ANGs, the cumbersome linker chemistry that reversibly converts the drug into a prodrug is obviated. Additionally, as the targeting mechanism is based on the OATP receptor, the surface ligand functionality on the surface of the nanogel is based on a simple anionic functionality, such as carboxylates. This greatly expands the repertoire of drug molecule that can be potentially used with this approach as well as providing less expensive manufacturing costs. The ability to encapsulate drug molecules or therapeutics and specifically target these compounds to the liver in a safe delivery system will be a major step forward in bringing this technology closer to the clinic. Future work can further explore these benefits of ANGs administration, such as evaluating the overall in vivo toxicity of the ANGs system and the nature and excretion of the polymer breakdown products upon in vivo administration. Current work with the ANGs system has been done in which animals have been dosed via intraperitoneal administration; this will need to be extended to subcutaneous administration for clinical adaptability (44, 45).

**Fig. 7.**
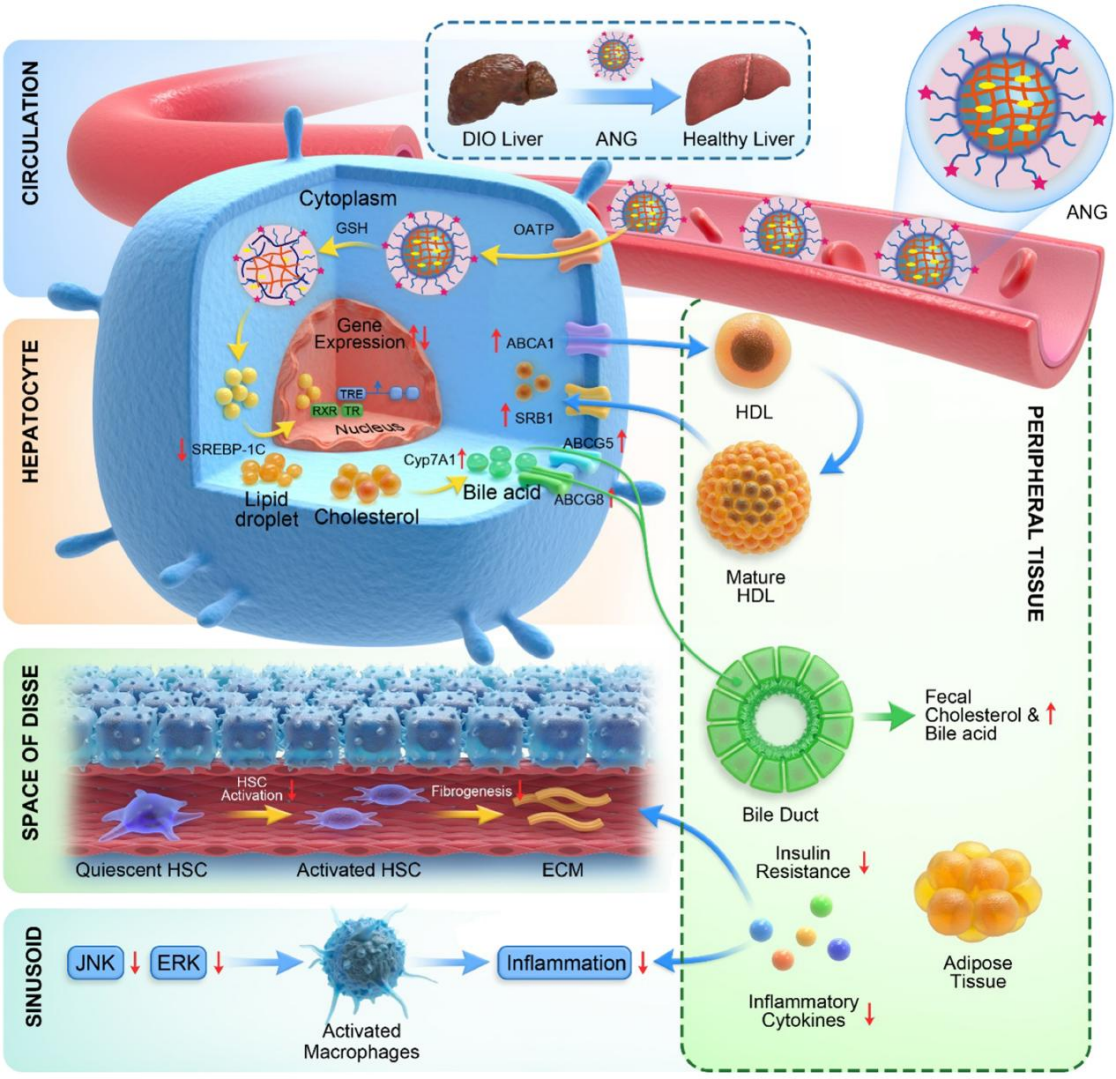
Schematic showing the mechanism by which CGS-ANG regulates metabolic dysfunction in mice. ANG sequesters CGS 26214 and efficiently targets hepatocytes by interacting with OATP. CGS 26214 is released after the cross-links of ANG break in reducing cytosolic environment. The drug exerts its action by binding to TRβ, which acts as a ligand-inducible transcription factor that interacts with TRE in the regulatory regions and modulates target gene expression. (i) CGS-ANG down-regulates lipogenesis genes such as SREBP-1c and decreases lipid accumulation. (ii) Genes involved in HDL reverse cholesterol pathway are stimulated, which leads to promoting the excretion of cholesterol and bile acids and the reduction of serum or liver cholesterol. (iii) Reduced obesity suppresses the oversecretion of adipocytokines and further inhibits the development of systemic insulin resistance and hepatic steatosis. The level of lipid moieties released by adipocytes is reduced and relieves the hepatocyte death and liver inflammation. In addition, JNK and ERK pathways are repressed, which stops the macrophages infiltration to liver tissues and activation of stellate cells to liver fibrosis.

In the DIO mouse model, our results suggest that CGS-ANG prevented, stopped, or reversed development of obesity and NAFLD under a synergistic mechanistic pathway. Although the preceding studies show that CGS 26214 exhibits unprecedented in vivo lipid-lowering potency, the mechanism for its action remains unknown. Herein, we summarize a possible mechanism by which CGS-ANG imparts its protective and functional properties (Fig. 7). Cross-linked nanogels with anionic ligand modification unleash the full therapeutic utility of thyromimetic drugs by selectively targeting their action profile to the liver. The thyromimetic drug exerts its action by binding to TRβ, which acts as a ligand-inducible transcription factor that interacts with TH response elements (TREs) in the regulatory regions and modulates target gene expression.

TH and their mimics decrease hepatic steatosis mainly by regulating fatty acid and cholesterol metabolism (2). In our study, SREBP-1c and its target genes, including fatty acid synthase (FASN) and acetyl CoA carboxylase (ACC) have been down-regulated due to the actions of CGS-ANG (Figs. 6B and S17), leading to repressed lipogenesis and liver triglyceride content (Fig. 4E). Although some reports suggest that TH increases the activity of hepatic lipases, lipophagy, and β-oxidation of fatty acids, which help fatty liver to reduce steatosis (2, 46–48), little changes were found here in genes involved in fat oxidation (CPT1a, CD36, and AOX) (Fig. S17). The reason may be the low lipid levels in livers with less steatosis. The large reduction of serum cholesterol induced by CGS-ANG was not accompanied by increased hepatic cholesterol (Fig. 4E), leading us to hypothesize that there is an increased flux of cholesterol through the high-density lipoprotein (HDL) reverse cholesterol pathway. The stimulated expression of eight different genes in the HDL reverse cholesterol pathway results in the enhanced transformation of cholesterol to bile acid and subsequent fecal excretion of these compounds. This may arise mainly from accelerating biliary excretion and limiting intestinal absorption of cholesterol and bile acid, while promoting fecal excretion (Figs. 6A and C, S16, and S18) (49–53). Reduced obesity and steatosis suppressed hepatic inflammation and fibrogenesis, which is shown in reduced mRNA expression of a range of hepatic inflammatory markers. Down-regulation of proinflammatory cytokines repressed JNK and ERK pathways (Figs. 6H and S22), which have been reported to drive macrophages to infiltrate liver tissues and activate fibrogenic genes to stimulate collagen production in stellate cells (54).

In summary, we report on a thyromimetic-loaded ANG system that exhibits specific liver-targeting ability, thus protecting the thyromimetic cargo from systemic exposure in vivo and enabling release on target. Treatment with CGS-ANG at all doses led to a complete reversal of body weight gain, liver weight gain, cholesterol levels, and significantly attenuated liver steatosis, inflammation, and fibrosis. The improvement in on-target efficacy of CGS 26214 by ANG encapsulation was statistically significant and robust. These data provide key mechanistic insights into the role of thyromimetics in lipogenesis, cholesterol metabolism, and inflammatory response. Unexpectedly, it demonstrates the potential of thyromimetics as an antiobesity agent, as CGS-ANG reversed body weight gain without systemic changes in T4 or TSH, providing a therapeutic solution for an enormous medical and societal health problem. A particularly noteworthy feature of the observed weight loss is that CGS-treated mice continued on the high-density diet with no loss of appetite.

In addition to weight loss, the pleotropic nature of the TH pathway suggests additional therapeutic areas of intervention, including NASH, type 2 diabetes, hyperlipidemia, and cardiovascular diseases. Even more broadly, the drug-encapsulated ANGs open up the possibility for nanoparticle-mediated pharmaceutical strategies for other liver-based diseases. For example, our nanogel system has demonstrated efficient protein encapsulation and intracellular redox-sensitive release (55). Given the large number of diseases involving the liver and the therapeutic proteins are the most important biologicals in terms of their clinical utility, it is possible that the ANG system can be used to devise biologics-based additional treatment strategies as well.

## Materials and methods

### Materials, cell culture, and animal care

Unless mentioned, all chemicals were used as received from Sigma-Aldrich. Pyridyl disulfide ethyl methacrylate (PDSEMA) was prepared using a previously reported procedure (20). Cy3 and Cy7 dyes were purchased from Lumiprobe. CGS 26214 was synthesized and purified by Keminntek Laboratories, India. T3 and KB2115 were purchased from Cayman.

The human hepatocellular carcinoma Hep G2 cells were purchased from ATCC. The cells were maintained in Dulbecco's modified Eagle's medium (DMEM/F-12, Gibco) supplemented with 10% fetal bovine serum (Gibco) and 1% antibiotics (penicillin and streptomycin, Gibco). The cells were cultured at 37°C in a humidified atmosphere of 5% CO_2_ and were subcultivated approximately every 2–3 days at 70–80% confluence at a split ratio of 1:3.

Male CD-1 mice at 8 weeks of age were purchased from Charles River Laboratories and male C57BL/6J mice at 6 or 10 weeks of age were purchased from the Jackson Laboratory. All of the mice were housed in a controlled environment (12-h light/dark cycle, 21 ± 2°C, humidity 50 ± 10%) and were permitted ad libitum access to water and rodent chow. The animal studies strictly followed the animal protocol approved by the Institutional Animal Care and Use Committee at the University of Massachusetts, Amherst and performed under the ethical guidelines for the use and care of animals.

### Synthesis of PEG:PDS random copolymers

The random copolymers containing PEG methacrylate and PDSEMA as side chain functionalities were synthesized using reversible addition-fragmentation chain-transfer (RAFT) polymerization as previously reported (20). Briefly, a mixture of PDSEMA (595.84 mg, 2.27 mmol), PEG methacrylate (average MW: 500, 500 mg, 1 mmol), 4-cyano-4-[(dodecyl-sulfanylthiocarbonyl)sulfanyl] pentanoic acid (27.05 mg, 0.067 mmol), and AIBN (2.2 mg, 0.0134 µmol) were dissolved in anhydrous THF (2.2 mL) and degassed by performing three freeze–pump–thaw cycles. The reaction mixture was sealed and then put into a preheated oil bath at 70°C for 10 h. To remove unreactive monomers, the resultant mixture was precipitated and washed in cold diethyl ether for several times to yield the random copolymer as a yellow gel.

^1^H-NMR spectra were recorded on a 400-MHz Bruker NMR spectrometer using the residual proton resonance of the solvent as the internal standard. Molecular weights of the polymers were estimated by GPC (Waters) using PMMA standard with a refractive index detector. The size of polymers was detected by DLS measurements using a Malvern Nano Zetasizer.

### Preparation of ANG and CGS-ANG

To prepare ANG with different sizes and ligand modification degrees, Cy3-linked PEG:PDS random copolymers (5 mg) were dissolved in distilled water (1 mL or 0.5 mL) for the final concentration of 5 or 10 mg/mL. Calculated amount of Na_2_CO_3_ was added to the micelle solutions and the mixtures were kept in 50°C oil bath for 6 h. Calculated amount of DTT (0.311 mg) was then added to the mixture and stirred for 24 h to generate NNG. NNGs were then modified with anionic ligands by adding calculated amount of 3-mercaptopropionic acid (0.429 mg) and stirred overnight.

All ANGs or CGS-ANGs used in the animal studies contained 28% cross-linking degree and 28% anionic ligand modification degree. For ANG, PEG:PDS random copolymers (50 mg) were dissolved in phosphate-buffered saline (PBS) buffer (5 mL) and calculated amount of DTT (3.11 mg) was then added to the mixture and stirred for 24 h to generate cross-linked drugless nanogels. For CGS-ANG, PEG:PDS random copolymers (50 mg) were dissolved in PBS buffer (5 mL) at first. Then, CGS 26214 (0.25 mg) dissolved in 150-µL THF was added into polymer solutions to form drug-encapsulated nanoassemblies. The mixture was stirred overnight at room temperature, open to the atmosphere to allow the organic solvent to evaporate. Calculated amount of DTT (3.11 mg) was added to the mixture and stirred for another 24 h to generate cross-linked drug-encapsulated nanogels. CGS-ANGs and ENGs were then modified with anionic ligands by adding calculated amount of 3-mercaptopropionic acid (4.29 mg) and stirred overnight.

The percentage of cross-linking was calculated by assuming that the formation of a single, cross-linking disulfide bond would require cleavage of two PDS units and produce two pyridothione molecules. The percentage of anionic ligand modification was calculated by assuming that formation of a single, cross-linking disulfide bond would require cleavage of one PDS unit and produce one pyridothione molecule. Cross-linking degree and ligand modification degree were determined by calculating the amount of byproduct 2-pyridinethione using its molar extinction coefficient (8.08 × 10^3^ M^–1^ cm^−1^ at 343 nm) by UV–visible (UV–vis) spectroscopy. The resultant ANG and CGS-ANG were purified by dialysis against PBS buffer (cutoff = 3,500 Da) for 3 days and sterile filtered through 0.22-µm Millipore polyvinylidene difluoride (PVDF) filters to remove free drugs. The size was then measured by DLS at 0.1 mg/mL. For TEM study, a 1 mg/mL sample was dropped onto Formvar-supported copper grids (Sigma-Aldrich) and was negatively stained by Nano-W (Nanoprobes) according to the manufacturer's instructions. Images were recorded using FEI Tecnai T12 instrument (Fisher Scientific) operated at 120 kV at a nominal magnification of 23,000×. At least eight locations on the TEM grid were examined.

To determine the drug loading capacity, CGS-ANG solution (0.5 mL, polymer concentration: 10 mg/mL) was subjected to cross-link breakage by adding extra amount of DTT (155 mg) and stirred for 8 h. Then, the solution was lyophilized for 8 h and the product was reconstituted in methanol for further analysis. Quantitative determination of CGS 26214 released from nanogels was conducted by a sensitive LC–MS/MS method (56). Briefly, each sample was hydrolyzed by mixing with 2 mL of a freshly prepared aqueous solution of 0.5 M ammonium hydroxide and kept on the bench for 60 min at room temperature. The hydrolysate was treated with 0.4 mL of glacial acetic acid to make the content slightly acidic (pH 4–5). The sample was dried in a Savant evaporator at room temperature. The residue was reconstituted in methanol and diluted 20 times for LC–MS/MS quantification.

### Cell uptake analysis

Hep G2 cells were seeded into 6-well plates at a density of 5 × 10^5^ cells/mL. After being cultured for 24 h, Cy3-ANG (0.2 mg/mL as equivalents) in FBS-free culture media was added into cells and incubated for 2 h. Cells were collected after trypsinization and the binding of ANG was detected by flow cytometry using the Cy3 fluorescence. For confocal laser scanning microscopy observation of nanogels internalization, Hep G2 cells were incubated with the Cy3-ANG for 2 h and were fixed in 4% formaldehyde for 15 min at room temperature. The polymerized/filamentous actin in cells was stained by CellMask Green Actin Tracking Stain (Invitrogen) at room temperature for 15 min, while the nucleus was stained by Hoechst (Invitrogen) at room temperature for 25 min. Finally, the samples were detected by confocal laser scanning microscope CrestV2 with 2xTIRF (Nikon).

### Pharmacokinetics and distribution analysis in vivo

For pharmacokinetics and biodistribution studies of ANGs, 8-week-old CD-1 mice were randomly divided into different treatment groups and intraperitoneally injected with Cy3-CNG and Cy3-ANG (80 mg/kg). The mice were anesthetized under carbon dioxide at specific time points after injection and sacrificed via cervical dislocation. The blood was collected into heparin blood collection tubes by cardiac puncture, followed by detaching the main organs. Blood samples were spun immediately to separate the red blood cells from the plasma (3,000 rpm × 15 min at 4°C). The plasma portion was transferred to fresh microcentrifuge tubes for analysis. The tissues were homogenized in PBS buffer and the homogenate was centrifuged at 10,000 rpm at 4°C for 20 min. The supernatants were collected, and Cy3-ANG signal was measured by SpectraMax iD5 (Molecular Devices)

For pharmacokinetics and biodistribution studies of CGS-ANG, 10-week-old C57BL/6J mice were randomly divided into different treatment groups and intraperitoneally injected with CGS 26214 free drug and CGS-ANG (3 mg/kg, CGS 26214 equivalent). The mice were anesthetized under carbon dioxide at specific time points after injection and sacrificed via cervical dislocation. The blood was collected by cardiac puncture, followed by detaching the main organs. Blood samples were spun immediately to separate the red blood cells from the plasma (3,000 rpm ×15 min at 4°C). The plasma portion was transferred to fresh microcentrifuge tubes for further analysis. The analytical method was developed as previously reported (46). Briefly, internal standard KB2115 was added to plasma or tissue homogenate and mixed by vortexing. The samples were extracted using C18 SPE cartridges (Bond Elute C18, 100 mg, 1-mL capacity, from Agilent Technologies) following the manufacturer's instructions. The elution was dried in Savant SPD131DDA SpeedVac Concentrator (Thermo Scientific) at room temperature and redissolved in distilled water by sonification. Extra amount of DTT was added to decross-link nanogels. Free CGS 26214 was hydrolyzed by mixing with a freshly prepared aqueous solution of 0.5 M ammonium hydroxide and kept on the bench for 60 min at room temperature. The hydrolysate was treated with glacial acetic acid to make the content slightly acidic (pH 4–5). The sample was dried in a Savant SPD131DDA SpeedVac Concentrator (Thermo Scientific) at room temperature. The residue was reconstituted in methanol for LC–MS/MS quantification.

For ex vivo fluorescence imaging studies, 10-week-old C57BL/6J mice were randomly divided into different treatment groups and intraperitoneally injected with Cy7-NNG and Cy7-ANG (80 mg/kg). The mice were anesthetized under carbon dioxide at specific time points after injection and sacrificed via cardiac puncture. Major organs were dissected and imaged ex vivo by IVIS SpectrumCT In Vivo Imaging System (PerkinElmer).

### Potent therapeutic effect of CGS-ANG in vivo

Ten-week-old C57BL/6J mice were randomly divided into different treatment groups and intraperitoneally injected with T3, CGS 26214 free drug, CGS-ANG (10, 20, and 60 µg/kg, T3/CGS 26214 equivalent) and ENG (ENG contained the same dose of ANG as the CGS-ANG group containing the highest dose) once daily for 7 days. Food was withdrawn 6 h before sacrifice. Mice were anesthetized under carbon dioxide and sacrificed via cervical dislocation at day 8. Plasma and main organs were collected, snap frozen, and stored at −80°C for lipid and gene expression analysis.

### DIO mice models and treatments

In the preventative study, 6-week-old C57BL/6J mice were permitted ad libitum access to water and either 10-kcal% fat control diet (CD, Cat# D09100304, Research Diets) or 40-kcal% fat, 20-kcal% fructose, and 2% cholesterol GAN diet (GAN diet, Cat# D09100310, Research Diets) for 12 weeks and then randomly assigned to nine groups with similar total body weights per group (except for control diet-feeding group) before the medical treatments: (i) HC: mice fed the CD diet and treated with vehicle; (ii) NC: mice fed the GAN diet and treated with vehicle; (iii) CGS-1: mice fed the GAN diet and treated with 10 µg/kg of CGS 26214 (suspended in PBS saline with 1% DMSO); (iv) CGS-2: mice fed the GAN diet and treated with 20 µg/kg of CGS 26214; (v) CGS-3: mice fed the GAN diet and treated with 60 µg/kg of CGS 26214; (vi) CNG-1: mice fed the GAN diet and treated with CGS-ANG loaded with CGS 26214 at a dose of 10 µg/kg; (vii) CNG-2: mice fed the GAN diet and treated with CGS-ANG loaded with CGS 26214 at a dose of 20 µg/kg; (viii) CNG-3: mice fed the GAN diet and treated with CGS-ANG loaded with CGS 26214 at a dose of 60 µg/kg; (ix) ENG: mice fed the GAN diet and treated with ENG with the same dose of nanogels with group (viii). All the treatments were injected intraperitoneally at the doses indicated once per day before the dark cycle of the day for 5 weeks.

In the therapeutic study, 6-week-old C57BL/6J mice were permitted ad libitum access to water and either 10-kcal% fat control diet (CD, Cat# D09100304, Research Diets) or 40-kcal% fat, 20-kcal% fructose, and 2% cholesterol GAN diet (GAN diet, Cat# D09100310, Research Diets) for 24 weeks and then randomly assigned to nine groups with similar total body weights per group (except for control diet-feeding group) before the medical treatments: (i) HC: mice fed the CD diet and treated with vehicle; (ii) NC: mice fed the GAN diet and treated with vehicle; (iii) CGS-1: mice fed the GAN diet and treated with 5 µg/kg of CGS 26214 (suspended in PBS saline with 1% DMSO); (iv) CGS-2: mice fed the GAN diet and treated with 10 µg/kg of CGS 26214; (v) CGS-3: mice fed the GAN diet and treated with 20 µg/kg of CGS 26214; (vi) CNG-1: mice fed the GAN diet and treated with CGS-ANG loaded with CGS 26214 at a dose of 5 µg/kg; (vii) CNG-2: mice fed the GAN diet and treated with CGS-ANG loaded with CGS 26214 at a dose of 10 µg/kg; (viii) CNG-3: mice fed the GAN diet and treated with CGS-ANG loaded with CGS 26214 at a dose of 20 µg/kg; (ix) ENG: mice fed the GAN diet and treated with ENG with the same dose of nanogels with group (viii). All the treatments were injected intraperitoneally at the doses indicated once per day before the dark cycle of the day for 5 weeks.

All diets were maintained for the entire duration of the studies. Body weight and food intake per cage were measured regularly during the study. Feces collections were done prior to sacrifice. Feces from individually housed mice were collected on each day of the 3-day feeding period. After each designated treatment period, all mice were fasted for 6 h and were sacrificed by cervical dislocation after gradual-fill CO_2_ anesthetization. Terminal blood samples were collected by cardiac puncture to isolate serum. Epididymal fat pads (EFPs), liver, and hearts were removed and weighed. Portions of livers were stored at −80°C or fixed in 4% paraformaldehyde solution (Sigma-Aldrich) for further analysis. Serum and other main organs were snap frozen and stored at −80°C.

### Lipids, bile acids, and serum chemistry analysis

Total liver and fecal lipids were extracted according to the Folch method (57). Briefly, lipids were extracted in a mixture of chloroform:methanol (2:1; v/v), dried in Savant SPD131DDA SpeedVac Concentrator (Thermo Scientific) and resuspended in isopropanol containing 1% Triton X-100 (Sigma-Aldrich). Total cholesterol and triglycerides were measured with Cholesterol Assay Kit (Abcam) and Triglyceride Assay Kit (Abcam) following the manufacturer's instruction. Serum HDL and LDL were measured by IDEXX BioAnalytics (Columbia, MO, USA). Total fecal bile acid was extracted and quantified using Mouse Total Bile Acids kit (Crystal Chem) according to manufacturer's instruction. Serum insulin, glucose (Crystal Chem), ALT, AST (Abcam), MCP-1, SAA, and E-selectin (Invitrogen) were measured by commercial assay kits. All enzyme-linked immunosorbent assay (ELISA) and colorimetric assays were performed according to the manufacturer's protocol.

### Reverse transcription quantitative real-time PCR

Total RNA was extracted from snap-frozen liver tissues using TRIzol reagent (Invitrogen). The cDNAs were synthesized with High-Capacity cDNA Reverse Transcription Kit (Applied Biosystems) according to the manufacturer's instructions. Gene expression analysis was performed with PowerUp SYBR Green Master Mix (Applied Biosystem) using ViiA 7 Real-Time PCR System (Applied Biosystem). Expression data were normalized to β-actin mRNA expression and fold change was calculated using 2^−ΔΔCt^ method. The specific primer sequences are listed in Table S3.

### Immunoblot analysis

The livers were lysed in RIPA buffer with protease and phosphatase inhibitors (Thermo Scientific) to extract liver proteins. Protein concentration was determined using bicinchoninic acid assay (Thermo Scientific). A 60-µg liver protein was separated by 12% (w/v) SDS–PAGE electrophoresis and transferred onto PVDF membranes. Membranes were blocked with Intercept (TBS) Blocking Buffer (LI-COR Biosciences) for 1 h at room temperature and then incubated with primary antibodies in Intercept T20 (TBS) Antibody Diluent (LI-COR Biosciences) overnight at 4°C on a rocking platform. The membranes were then incubated with IRDye 800CW Goat anti-Rabbit IgG or IRDye 680RD Goat anti-Mouse IgG secondary antibody (LI-COR Biosciences). The blots were visualized with infrared dye, and the infrared fluorescence of the blots was detected using Odyssey CLx Infrared Imaging System (LI-COR Biosciences). The following primary antibodies were used: SAPK/JNK antibody (9252; CST), Phospho-SAPK/JNK (Thr183/Tyr185) (G9) antibody (9255; CST), p44/42 MAPK (Erk1/2) (3A7) antibody (9107; CST), Phospho-p44/42 MAPK (Erk1/2) (Thr202/Tyr204) antibody (9101; CST), and GAPDH (14C10) antibody (2118; CST).

### Histology and immunohistochemistry

Sections of fresh livers from the left lateral lobes were fixed in 4% paraformaldehyde (Sigma-Aldrich) for 48–72 h and then stored in 75% (v/v) ethanol (Sigma-Aldrich) for embedding in paraffin or transferred to 30% (w/v) sucrose (Sigma-Aldrich) for embedding in optimal cutting temperature (OCT) compound. The liver samples were subsequently embedded, sectioned, and stained with H&E, MT, and oil red O by iHisto (Salem, MA). NAFLD activity was scored blindly by board-certified pathologists in H&E and MT stained cross-sections using a modified Kleiner scoring system that is applicable to all stages of NAFLD etiology by IDEXX BioAnalytics (Columbia, MO, USA). Immunohistochemical detection of F4/80 and α-SMA was performed on paraffin-embedded liver sections by HistoWiz, Inc. (Brooklyn, NY, USA). Positive areas were quantified from three randomly chosen fields per liver section from individual mice with six mice per group using ImageJ.

### Statistics

The results of all the experiments were generated from at least three independent replicates unless noted otherwise in the figure legend. All data are shown as the mean ± SD or mean ± SEM. Statistical analysis was evaluated using GraphPad Prism (v.8.0). Sample size was estimated based on previous experience, sample availability, and previous reported studies. No data were excluded from the data analysis. For statistical comparisons, unpaired two-tailed Student’S *t*-test was used to compare two groups, and one-way ANOVA with Tukey's or Dunnett's T3 post hoc analysis was used for multiple-group analyses. The differences between experimental groups and control groups were considered to be statistically significant at *P* < 0.05.

## Supplementary Material

pgad252_Supplementary_Data

## Data Availability

The data that support the findings of this study are available in the supporting information of this article.
