# Supplementary material for: Conferring liver selectivity to a thyromimetic using a novel nanoparticle increases therapeutic efficacy in a diet-induced obesity animal model
Source: PNAS Nexus. 2023 Aug 29;2(8):pgad252. doi: 10.1093/pnasnexus/pgad252 (PMC10465086; doi:10.1093/pnasnexus/pgad252)
Supplement: pgad252_Supplementary_Data [file pgad252_supplementary_data.pdf]

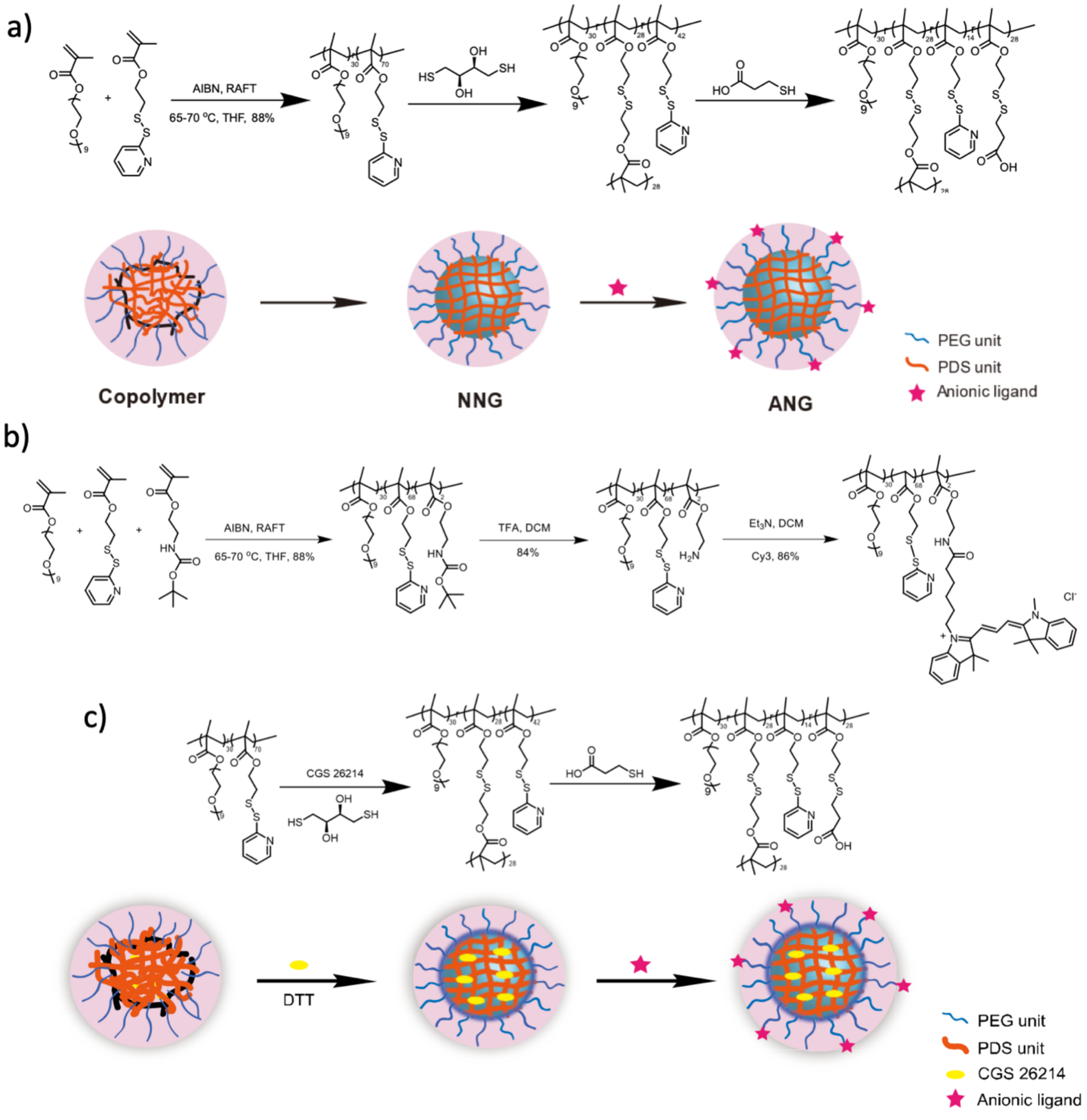

Scheme S1. Schematic illustration of the synthetic route of anionic nanogel (ANG) and CGS-encapsulated ANG. a, Synthesis of ANG. Amphiphilic random copolymer containing polyethylene glycol (PEG) methacrylate and pyridinyldisulfide (PDS) ethyl methacrylate were used as precursor and nanogels were formed by crosslinking the PDS groups. Anionic nanogels can be readily afforded by further functionalization with 3- mercaptopropionic acid. **b,** Synthesis of Cy3-linked copolymers for nanogel preparation. **c,** Preparation of CGS 26214-encapsulated ANG. CGS 26214 dissolved in acetone was added to micelle solutions and the mixture was stirred overnight at room temperature, open to the atmosphere allowing the organic solvent to evaporate. A measured amount of DTT was then added for cross-linking. Excess unencapsulated CGS 26214 was removed by filtration.

	NC	HC	CNG-1	CNG-2	CNG-3	CGS-1	CGS-2	CGS-3	ENG
**Body weight (g)**	44.2±1.0$^{\#\#\#\#}$	32.5±0.8	31.9±1.3***	33.5±1.1***	32.8±0.6***	39.7±2.1$^{\#\#}$	41.4±1.3$^{\#\#\#\#}$	39.0±1.3$^{\#,*}$	40.9±1.1$^{\#\#}$
**Liver weight (g)**	3.04±0.19$^{\#\#\#\#}$	1.18±0.06	1.43±0.14***	1.49±0.09***	1.59±0.11***	2.19±0.15$^{\#\#\#\#,**}$	2.00±0.17$^{\#\#,***}$	1.84±0.11$^{\#,***}$	2.59±0.17$^{\#\#\#\#}$
**LW/BW (%)**	6.77±0.30$^{\#\#\#\#}$	3.62±0.10	4.45±0.32***	4.44±0.21***	4.84±0.34$^{\#\#,***}$	5.24±0.28$^{\#\#\#\#,***}$	4.78±0.27$^{\#,***}$	4.67±0.16***	6.28±0.28$^{\#\#\#\#}$
**Heart weight (g)**	0.146±0.004	0.154±0.007	0.158±0.008	0.152±0.007	0.157±0.005	0.158±0.006	0.146±0.004	0.177±0.008**	0.151±0.007
**HW/BW (%)**	0.333±0.014$^{\#\#\#\#}$	0.471±0.017	0.500±0.022***	0.459±0.028***	0.480±0.016***	0.383±0.019	0.358±0.016$^{\#\#}$	0.458±0.024***	0.377±0.026
**Epididymal Fat Pads (g)**	2.39±0.12$^{\#\#\#\#}$	1.16±0.09	0.83±0.08***	1.08±0.13***	0.91±0.10***	2.44±0.14$^{\#\#\#\#}$	2.31+±0.16$^{\#\#\#\#}$	1.76±0.21*	2.21±0.19$^{\#\#\#\#}$
**EW/BW (%)**	5.37±0.24$^{\#\#\#\#}$	3.53±0.23	2.55±0.16***	3.17±0.30***	2.75±0.31***	5.91±0.33$^{\#\#\#\#}$	5.52±0.27$^{\#\#\#\#}$	4.42±0.43	5.35±0.34$^{\#\#}$

	NC	HC	CNG-1	CNG-2	CNG-3	CGS-1	CGS-2	CGS-3	ENG
**Body weight (g)**	51.4±1.1$^{\#\#\#}$	37.0±0.8	39.3±0.9***	37.0±1.0***	35.8±1.8***	46.5±1.1$^{\#\#\#,*}$	45.8±1.1$^{\#\#\#,**}$	44.6±0.9$^{\#\#\#,***}$	52.1±1.0$^{\#\#\#}$
**Liver weight (g)**	4.69±0.23$^{\#\#\#}$	1.65±0.08	2.15±0.15***	1.77±0.12***	1.78±0.16***	3.06±0.19$^{\#\#\#,***}$	2.97±0.19$^{\#\#\#,***}$	2.89±0.17$^{\#\#\#,***}$	4.95±0.16$^{\#\#\#}$
**LW/BW (%)**	9.03±0.27$^{\#\#\#}$	4.46±0.15	5.50±0.12***	4.77±0.28***	4.96±0.20$^{\#\#,***}$	6.53±0.30$^{\#\#\#,***}$	6.44±0.28$^{\#\#\#,***}$	6.46±0.28$^{\#\#\#,***}$	9.49±0.17$^{\#\#\#}$
**Heart weight (g)**	0.171±0.004$^{\#}$	0.147±0.007	0.165±0.008	0.151±0.005	0.148±0.005	0.167±0.007	0.152±0.005	0.171±0.008	0.189±0.010$^{\#\#\#}$
**HW/BW (%)**	0.334±0.008$^{\#}$	0.402±0.024	0.430±0.015***	0.408±0.013**	0.422±0.022*	0.359±0.014	0.332±0.010	0.383±0.018	0.365±0.020
**Epididymal Fat Pads (g)**	2.52±0.06$^{\#\#\#}$	1.50±0.09	1.66±0.10***	1.51±0.11***	1.27±0.16***	2.38±0.10$^{\#\#\#}$	2.19+±0.10$^{\#\#\#}$	2.17±0.09$^{\#\#\#}$	2.49±0.11$^{\#\#\#}$
**EW/BW (%)**	4.94±0.15$^{\#\#}$	4.01±0.18	4.21±0.16	4.04±0.21*	3.45±0.37***	5.14±0.21$^{\#\#}$	4.77±0.13	4.89±0.21	4.78±0.18$^{\#\#}$

Table S1. Selected parameters of healthy mice treated with vehicle (HC) or NASH mice treated with vehicle (NC), CGS 26214 of three doses (CGS-1,2,3), CGS-ANG of three doses (CNG-1,2,3) and ENG on in five weeks. Upper: preventing study; Lower: therapeutic study. All data are shown as mean ± s.e.m. n= 8-20 biologically independent mice per group. Statistical significance was calculated via Ordinary one-way ANOVA with Tukey's multiple comparison test. *P< 0.05; **P< 0.01; ***P< 0.001; ****P< 0.0001 compared to NC control. $^{\#}$P< 0.05; $^{\#\#}$P< 0.01; $^{\#\#\#}$P< 0.001; $^{\#\#\#\#}$P< 0.0001 compared to HC control.

	NC	HC	CNG-1	CNG-2	CNG-3	CGS-1	CGS-2	CGS-3	ENG
Cholesterol (mg/dL)	445.2±16.4	189.5±8.1****	208.0±9.1****	130.9±11.6****	145.1±20.9****	278.9±20.2****	236.8±16.6****	196.9±10.1****	374.8±18.9
HDL Cholesterol (mg/dL)	135.3±2.1	86.0±3.2****	101.5±5,3****	66.0±5.7****	65.6±6.6****	116±4.4	113.4±5.5*	96.3±3.9****	126.5±3.8
LDL Cholesterol (mg/dL)	52.4±3.5	12.7±1.1	12.8±0.7	<7	<7	18.4±2.3	15.3±1.1	11.4±1.2	40.8±3.0

Table S2. Selected parameters of healthy mice treated with vehicle (HC) or NASH mice treated with vehicle (NC), CGS 26214 of three doses (CGS-1,2,3), CGS-ANG of three doses (CNG-1,2,3) and ENG on in five weeks. All data are shown as mean ± s.e.m. n= 8-10 biologically independent mice per group. Statistical significance was calculated via Ordinary one-way ANOVA with Tukey's multiple comparison test. *P< 0.05; **P< 0.01; ***P< 0.001; ****P< 0.0001 compared to NC control.

Name	Sequences
ABCA1 FWD	GCTTGTTGGCCTCAGTTAAGG
ABCA1 REV	GTAGCTCAGGCGTACAGAGAT
ApoA1 FWD	GGCACGTATGGCAGCAAGAT
ApoA1 REV	CCAAGGAGGAGGATTCAAACTG
SR-B1 FWD	AAACAGGGAAGATCGAGCCAG
SR-B1 REV	GGTCTGACCAAGCTATCAGGTT
HMGCR FWD	AGCTTGCCCGAATTGTATGTG
HMGCR REV	TCTGTTGTGAACCATGTGACTTC
Cyp7A1 FWD	GCTGTGGTAGTGAGCTGTTG
Cyp7A1 REV	GTTGTCCAAAGGAGGTTCACC
Cyp8B1 FWD	CCTCTGGACAAGGGTTTTGTG
Cyp8B1 REV	GCACCGTGAAGACATCCCC
Cyp4A14 FWD	TGAATTGCTGCCAGATCCCAC
Cyp4A14 REV	GTTCAGTGGCTGGTCAGAGTT
ABCG5 FWD	AGGGCCTCACATCAACAGAG
ABCG5 REV	GCTGACGCTGTAGGACACAT
ABCG8 FWD	CTGTGGAATGGGACTGTACTTC
ABCG8 REV	GTTGGACTGACCACTGTAGGT
SREBP-1C FWD	GGAGCCATGGATTGCACATT
SREBP-1C REV	GGCCCGGGAAGTCACTGT
CD36 FWD	GTGCAAAACCCAGATGACGT
CD36 REV	TCCAACAGACAGTGAAGGCT
CPT1A FWD	ATCGTGGTGGTGGGTGTGATAT
CPT1A REV	ACGCCACTCACGATGTTCTTC
ACC1 FWD	TGGAGCTAAACCAGCACTCC
ACC1 REV	GCCAAACCATCCTGTAAGCC
AOX FWD	TGTCATTCCTACCAACTGTC
AOX REV	CCATCTTCTCAACTAACACTC
FASN FWD	TGGGTTCTAGCCAGCAGAGT
FASN REV	ACCACCAGAGACCGTTATGC
LDLR FWD	TGACTCAGACGAACAAGGCTG
LDLR REV	ATCTAGGCAATCTCGGTCTCC
SREBP2 FWD	GCAGCAACGGGACCATTCT
SREBP2 REV	CCCCATGACTAAGTCCTTCAACT
ApoB FWD	TCACCATTTGCCCTCAACCTAA
ApoB REV	GAAGGCTCTTTGGAAGTGTAAAC
Dio1 FWD	CCACCTTCTTCAGCATCC
Dio1 REV	AGTCATCTACGAGTCTCTTG
Me1 FWD	GCCAGAGGATGTCGTCAAGG

Me1 REV	ATTACAGCCAAGGTCTCCCAAG
MCP FWD	GGCTCAGCCAGATGCAGTTAA
MCP REV	AGCCTACTCATTGGGATCATCTT
TNFα FWD	GTAGCCCACGTCGTAGCAAAC
TNFαREV	AGTTGGTTGTCTTTGAGATCCATG
F480 FWD	TCAAGGACACGAGGT TGCTGA
F480 REV	CCAAGGGGCCAATCTGGAA
CD11b FWD	TCAGAGAATGTCCTCAGCAG
CD11b REV	TGAGACAAACTCCTTCATCTTC
CD68 FWD	CACTTCGGGCCATGTTTCTC
CD68 REV	AGGACCAGGCCAATGATGAG
IL1a FWD	CGCTTGAGTCGGCAAAGAAA
IL1a REV	TGATACTGTCACCCGGCTCT
IL1b FWD	ACCCTGCAGCTGGAGAGTGT
IL1b REV	TTGACTTCTATCTTGTTGAAGACAAACC
COL1A1 FWD	TGTTCAGCTTTGTGGACCTC
COL1A1 REV	GCAGCTGACTTCAGGGATGT
αSMA FWD	ACTGGGACGACATGGAAAAG
αSMA REV	GTGCCTCTGTCAGCAGTGTC
β-Actin FWD	GGCTGTATTCCCCTCCATCG
β-Actin REV	CCAGTTGGTAACAATGCCATGT

Table S3. List of genes examined by real-time PCR and the primer sequences used.

a

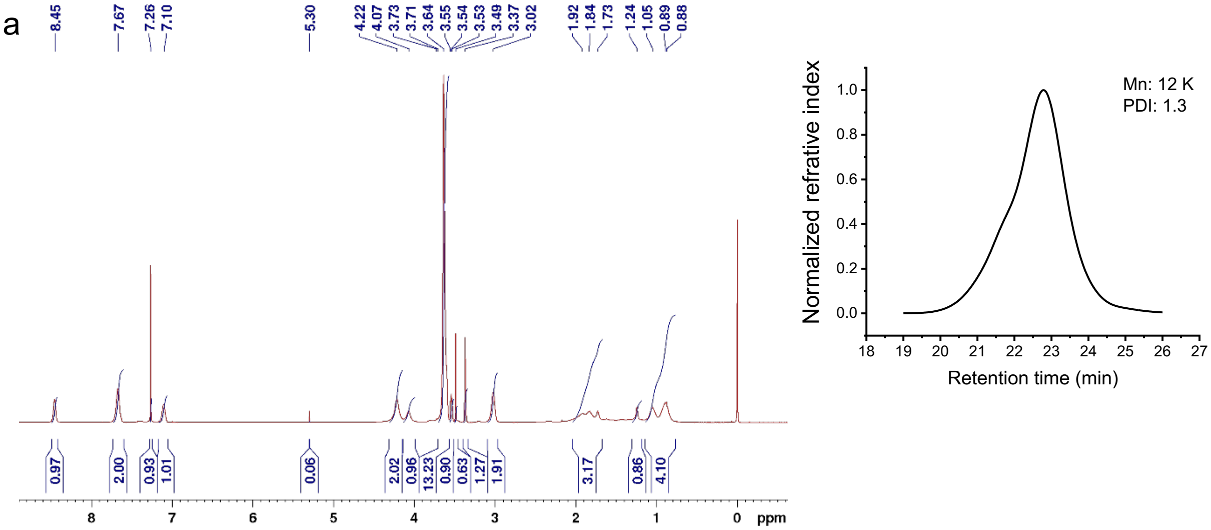

b

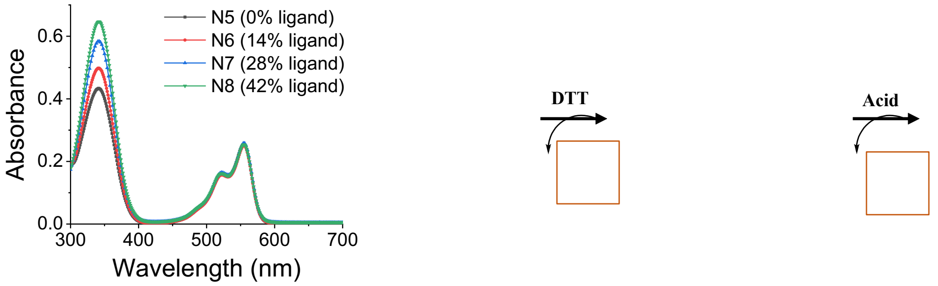

Figure S1. Characterization of PEG: PDS copolymers and ANG system. a, 1H nuclear magnetic resonance (1H-NMR) spectra and GPC trace of PEG: PDS random copolymers; **b,** Absorption spectra of pyridothione in UV-vis. The progress of cross-linking reaction and anionic ligand modification of nanogels was conveniently monitored by release of the pyridothione byproduct through tracing its characteristic absorption at 343 nm.

a

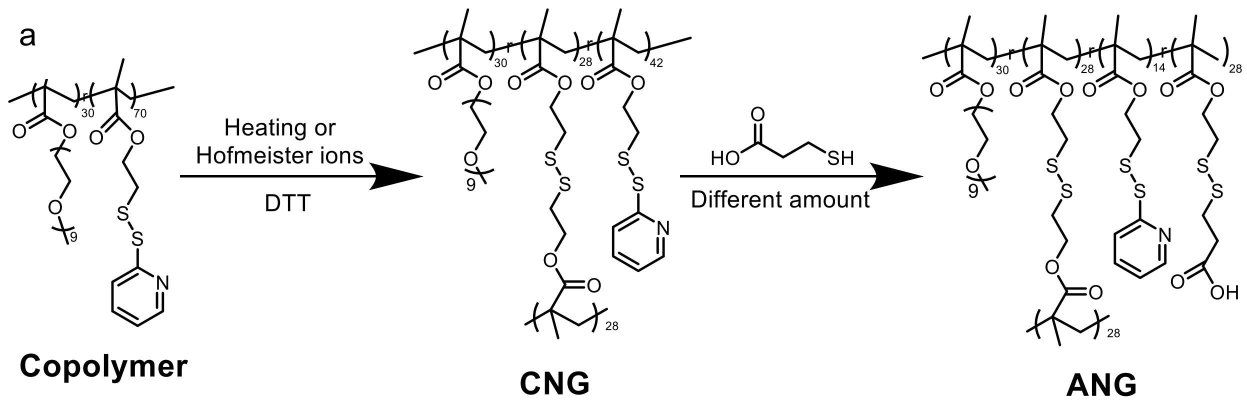

Nanogel	Preparation Condition
N1	10 mg/mL polymers, 50 ºC, 2 mM Na_2CO_3
N2	5 mg/mL polymers, 50 ºC, 6 mM Na_2CO_3
N3	10 mg/mL polymers, 50 ºC, 6 mM Na_2CO_3
N4	10 mg/mL polymers, 50 ºC, 10 mM Na_2CO_3

b

Nanogels	Hydrodynamic size (nm)	PDI	Ligand modification (%)
N1	29.5 ± 1.0	0.132 ± 0.003	28.6 ± 0.6
N2	47.7 ± 0.7	0.143 ± 0.011	27.4 ± 0.3
N3	69.7 ± 0.7	0.102 ± 0.006	27.2 ± 0.6
N4	104.8 ± 1.0	0.130 ± 0.005	26.3 ± 0.6
N5	34.2 ± 1.6	0.216 ±0.013	0
N6	31.3 ± 0.9	0.226 ± 0.026	13.9 ± 0.4
N7	34.3 ± 0.4	0.250 ± 0.017	32.1 ± 0.4
N8	34.3 ± 0.6	0.324 ± 0.030	43.4 ± 0.5

c

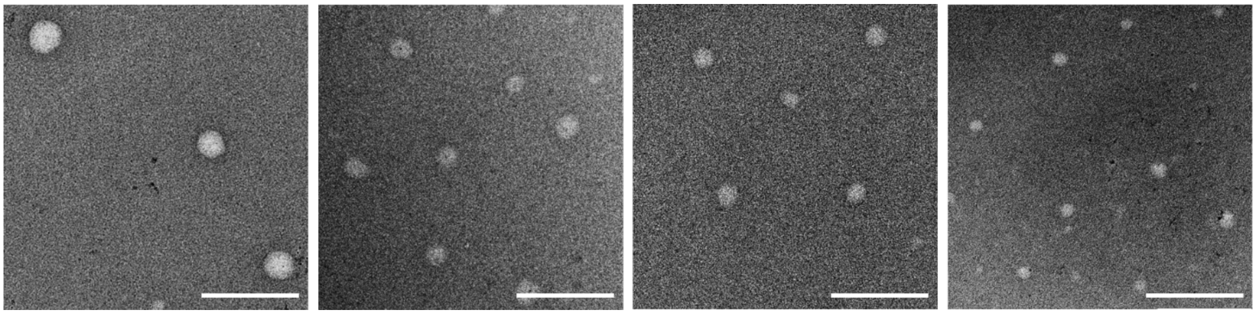

Figure S2. a, Synthesis scheme and preparation condition of nanogels with different size and anionic modification; **b,** Characterization of size and anionic ligand modification of nanogels**;** **c,** Negatively stained transmission electron microscopy images of ANG with different size (left to right: N1, N2, N3, N4, scale bar, 500 nm)

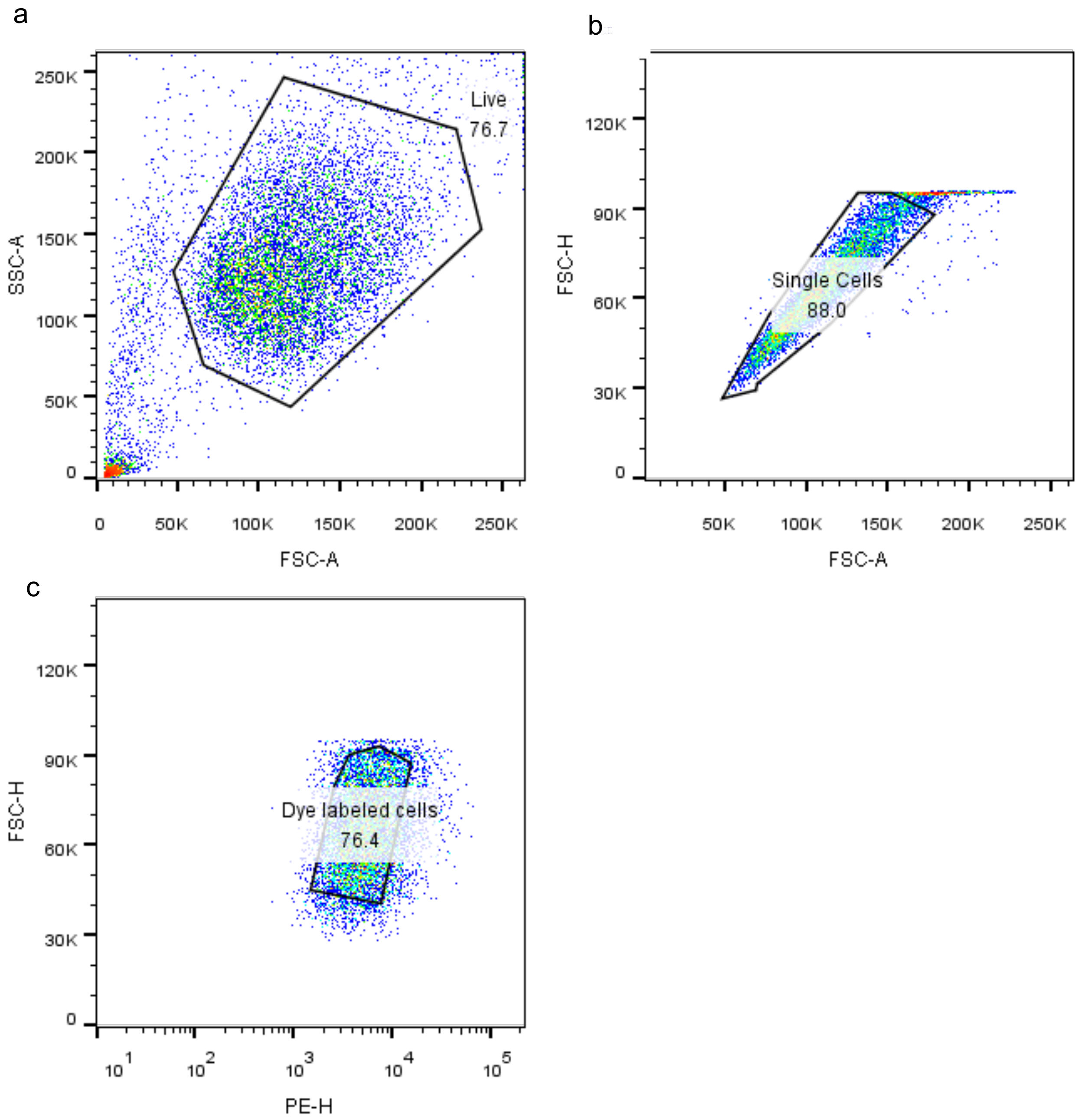

Figure S3. Gating strategy for flow cytometry. **a,** General population plotted in FSC, SSC gate and cells discriminated from debris based event size and distribution. **b,** Cells were gated on FSC-A and FSC-H plots to select single cells. **c,** Dye-labeled cells were determined as PE positive cells in a PE vs FSC plot.

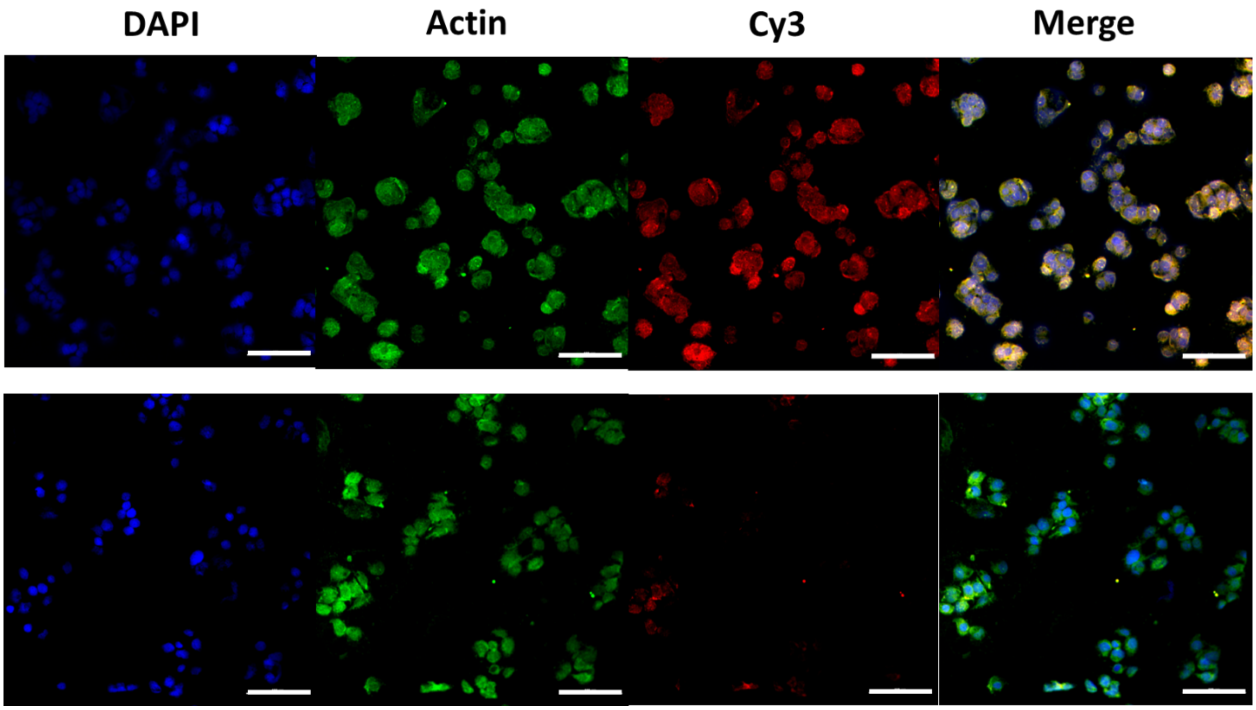

Figure S4. Representative confocal laser scanning microscope images of Cy3–ANG (30 nm, 40% ligand modification) colocalized with actin cytoskeleton (Upper panels). Cy3-NNG showed much lower signal than ANG group and mainly retained in cell periphery (Lower panels). Blue, nucleus; red, nanogels; green, actin; scale bar, 100 μm.

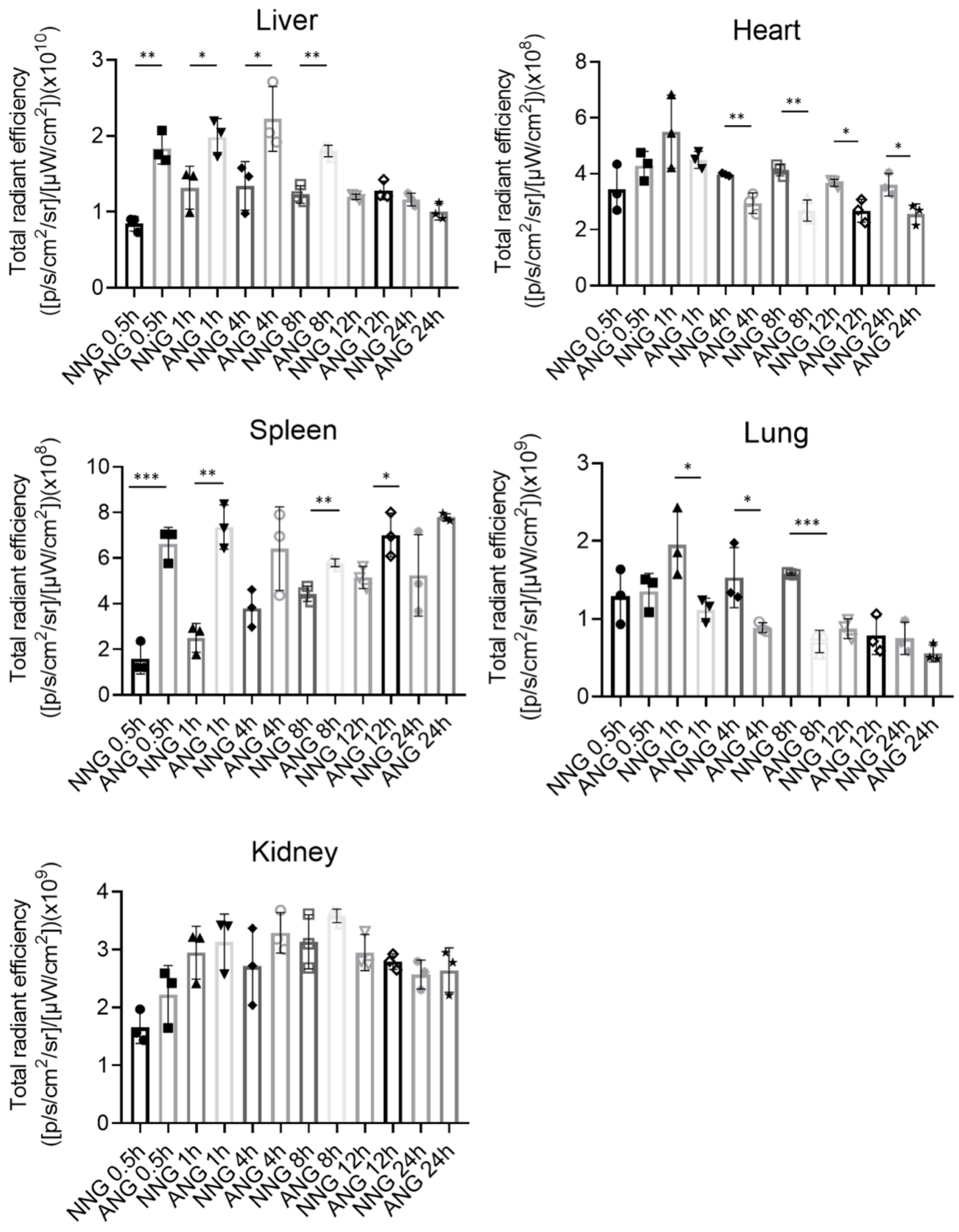

Figure S5. Quantitative fluorescence intensity of Cy7-NG in major organs from C57BL/6J mice receiving nanogels over time post-injection in ex vivo IVIS imaging study. Data are shown as the mean ± s.d. of n= 3 biologically independent mice per time point. Scale bar, red to blue, signal intensity high to low. Statistical significance was calculated via two-tailed Student's t-test. *P< 0.05; **P< 0.01; ***P< 0.001; ****P< 0.0001.

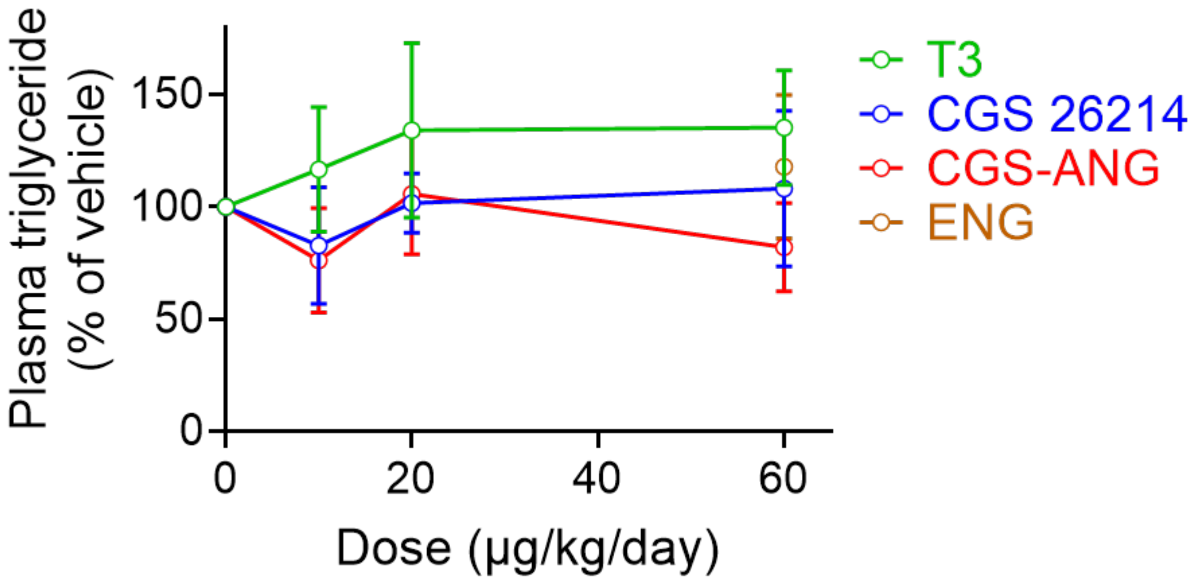

Figure S6. Effects of increasing dose of CGS-ANG, CGS 26214, T3 and ENG on total plasma triglyceride of normal mice. ENG is only shown at the 60 µg/kg/day, equivalent to the highest ANG in CGS-ANG dose. Results are expressed as fold change relative to the PBS vehicle treated control**.** Data are shown as mean ± s.e.m. (n= 6 biologically independent mice per group). Statistical significance was calculated via Ordinary one-way ANOVA with Tukey's multiple comparison test.

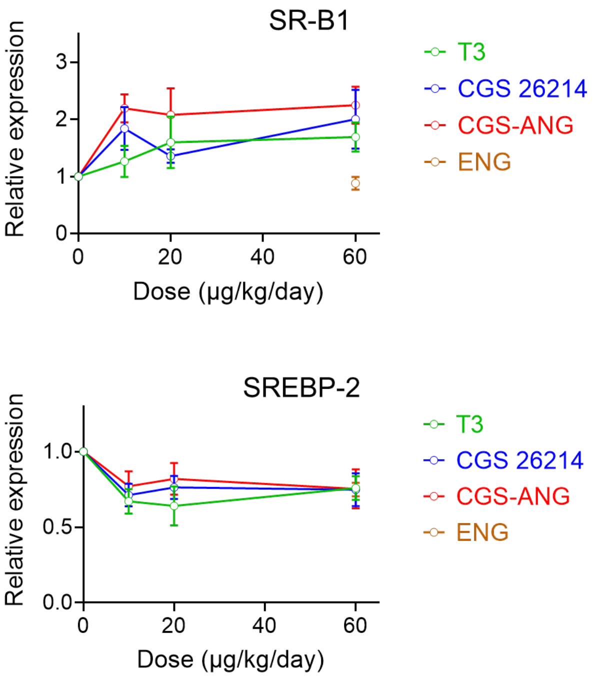

Figure S7. Relative mRNA expression of SR-B1, LDLR and SREBP-2 in livers from the PBS, CGS-ANG, CGS 26214, T3 and ENG treatment groups. ENG is only shown at the 60 µg/kg/day, equivalent to the highest ANG in CGS-ANG dose. All data are shown as mean ± s.e.m. (n= 6 biologically independent mice per group). Statistical significance was calculated via Ordinary one-way ANOVA with Tukey's multiple comparison test.

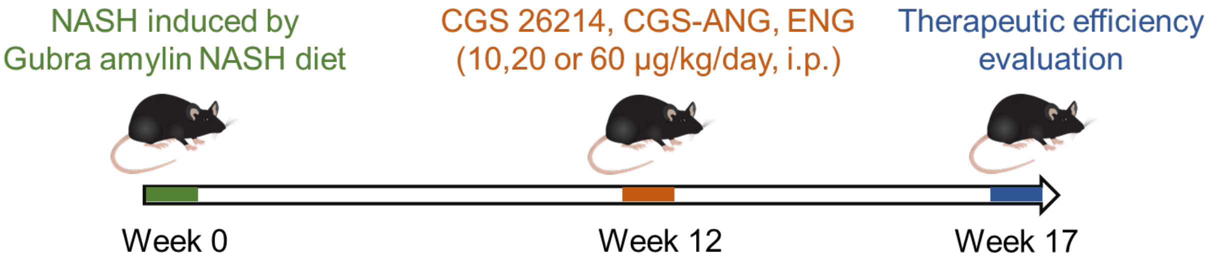

Figure S8. Schematic illustration of protocol for mild NASH disease establishment in C57BL/6J mice and preventing study of CGS-ANG. Twelve weeks after GAN diet feeding, mice were injected intraperitoneally daily by CGS 26214, CGS-ANG with three doses (dose 1,2,3 – CGS 10,20,60 μg/kg/day) and ENG for five weeks. CGS-ANG loaded with CGS 26214 at the dose that is the same as corresponding CGS 26214 groups. ENG contained the same dose of ANG with the highest dose CGS-ANG group.

a

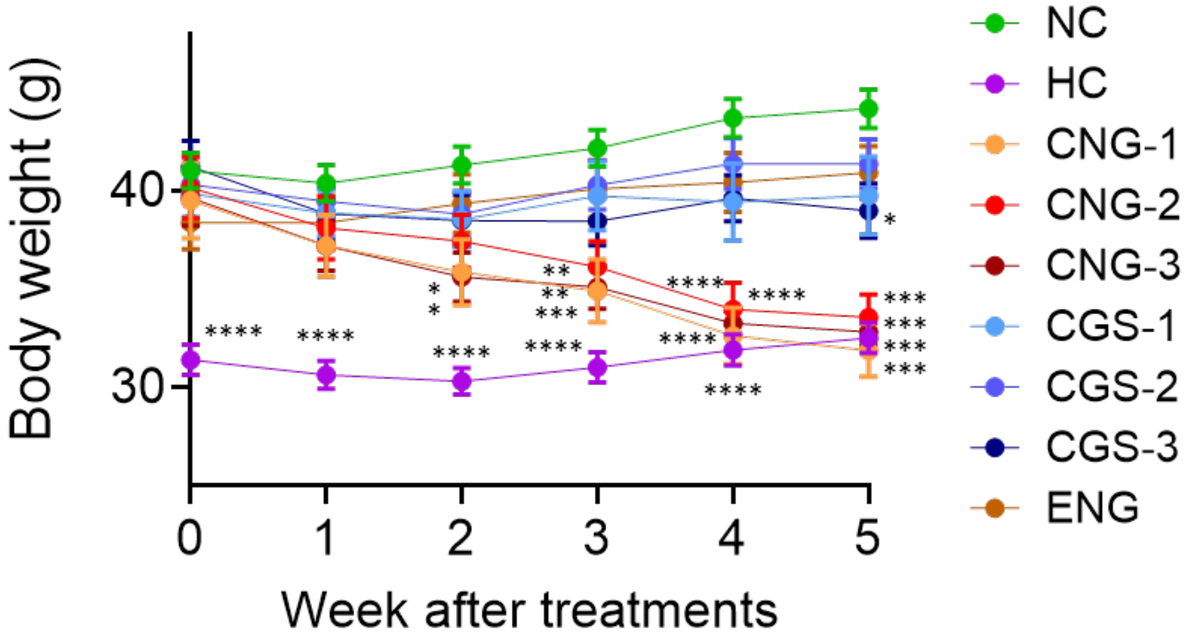

b

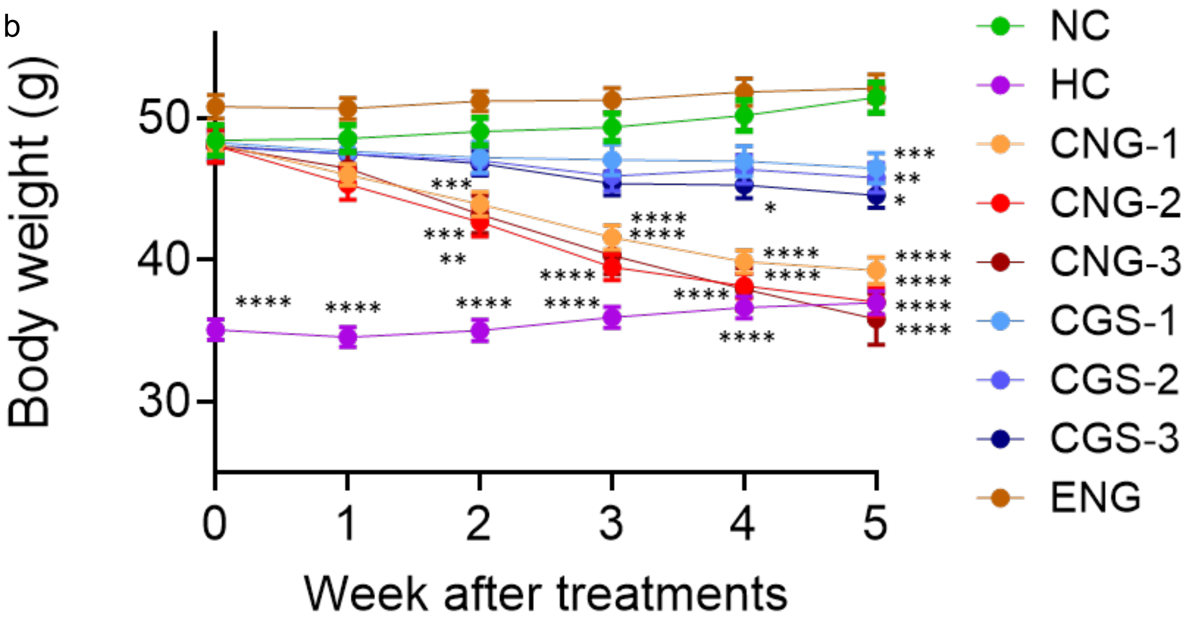

Figure S9. Body weight changes of healthy mice treated with vehicle (HC) or NASH mice treated with vehicle (NC), CGS 26214 of three doses (CGS-1,2,3), CGS-ANG of three doses (CNG-1,2,3) and ENG in five weeks in preventing study **(a)** and therapeutic study **(b)**. All data are shown as mean ± s.e.m. n= 8-20 biologically independent mice per group. Statistical significance was calculated via Ordinary one-way ANOVA with Tukey's multiple comparison test. *P< 0.05; **P< 0.01; ***P< 0.001; ****P< 0.0001 compared to NC control.

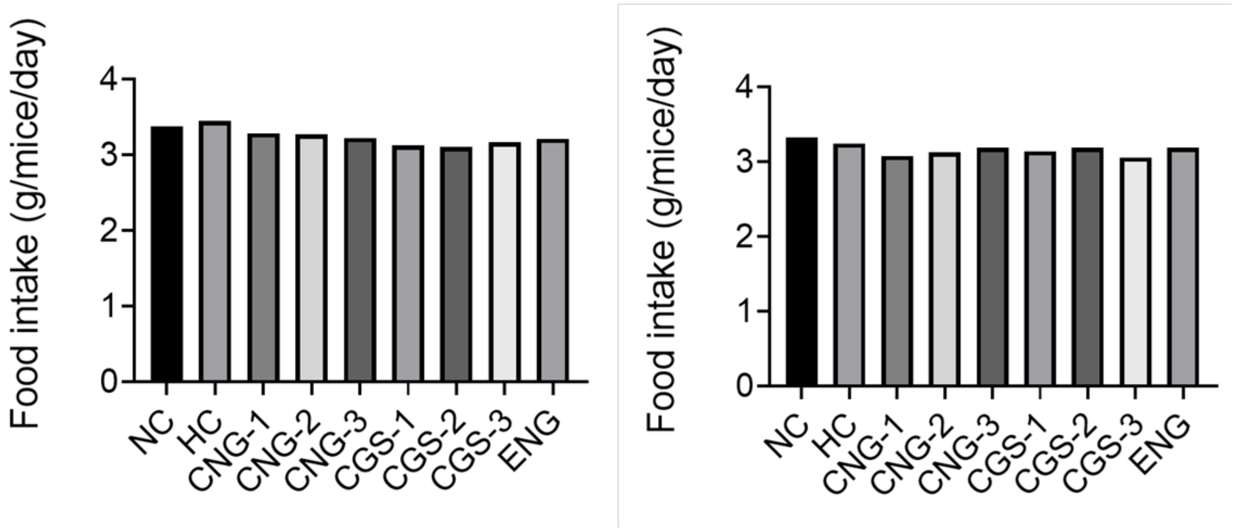

Figure S10. Daily food intake of healthy mice treated with vehicle (HC) or NASH mice treated with vehicle (NC), CGS 26214 of three doses (CGS-1,2,3), CGS-ANG of three doses (CNG-1,2,3) and ENG in 24 hours in preventing study **(a)** and therapeutic study **(b)**. Food intake was determined from total food consumption of mice every cage during a 24 h period so the statistical variation cannot be reported.

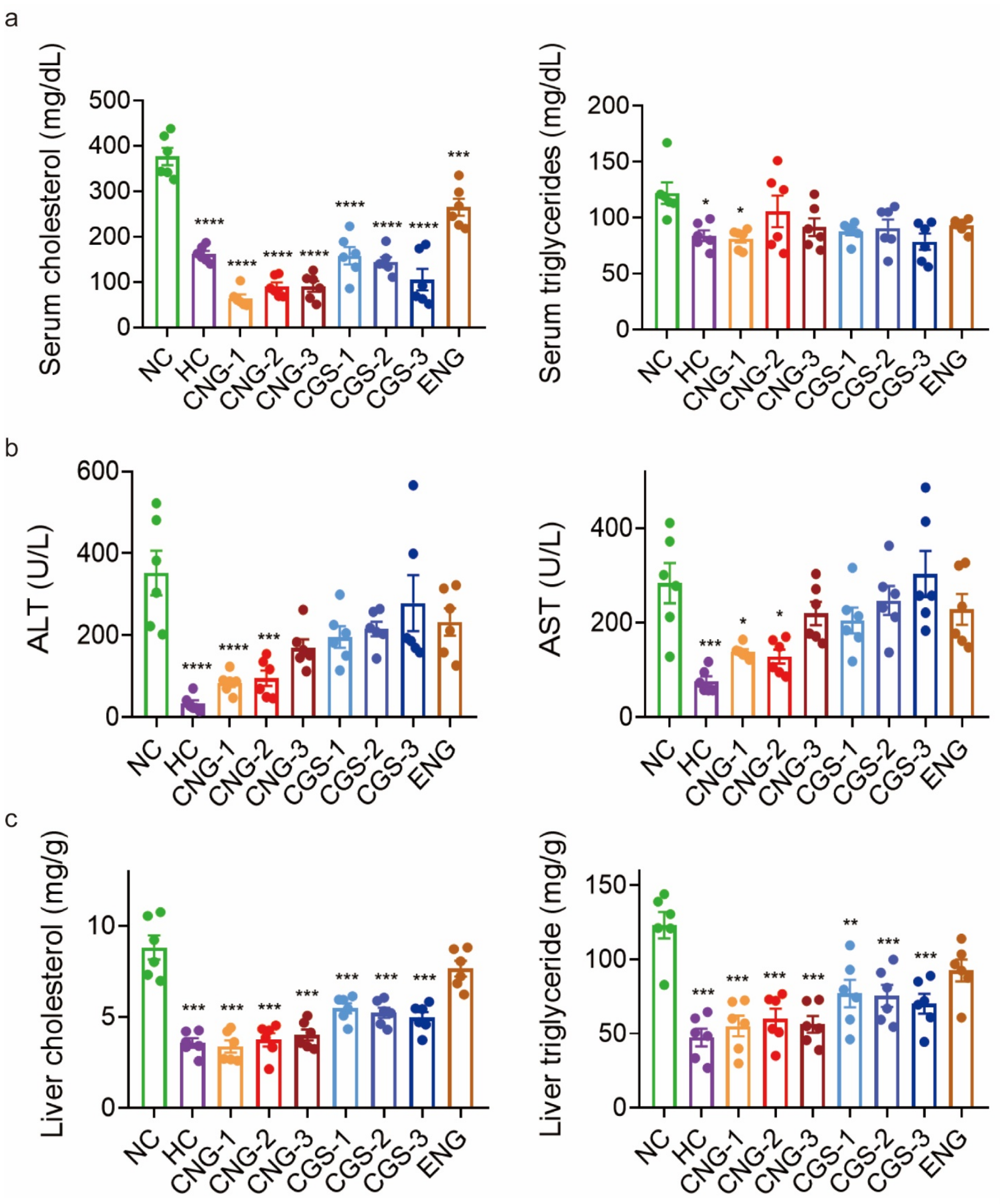

Figure S11. Effects of vehicle, CGS 26214 of three doses (CGS-1,2,3), CGS-ANG of three doses (CNG-1,2,3) and ENG on total serum cholesterol and triglyceride **(a)**, serum ALT and AST **(b)** and total liver cholesterol and triglyceride **(c)** of mice in five weeks in preventing study. All data are shown as mean ± s.e.m. n= 8-10 biologically independent mice per group. Statistical significance was calculated via Ordinary one-way ANOVA with Tukey's multiple comparison test. *P< 0.05; **P< 0.01; ***P< 0.001; ****P< 0.0001 compared to NC. control.

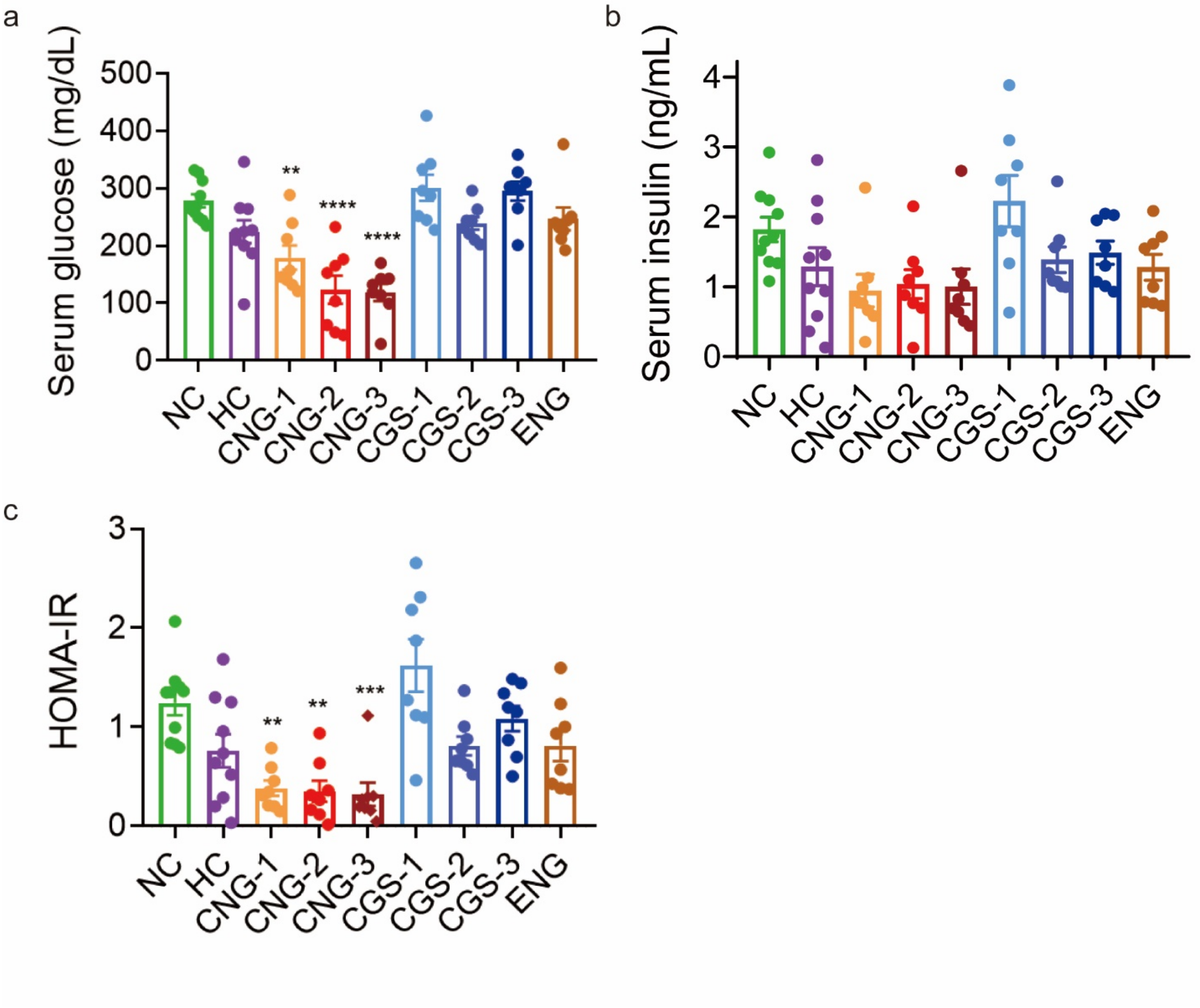

Figure S12. Effects of vehicle, CGS 26214 of three doses (CGS-1,2,3), CGS-ANG of three doses (CNG-1,2,3) and ENG on serum glucose **(a)**, insulin **(b)**, homeostasis model of assessment-insulin resistance (HOMA-IR) levels **(c)** of mice in five weeks in therapeutic study. All data are shown as mean ± s.e.m. n= 8-10 biologically independent mice per group. Statistical significance was calculated via Ordinary one-way ANOVA with Tukey's multiple comparison test. *P< 0.05; **P< 0.01; ***P< 0.001; ****P< 0.0001 compared to NC. control.

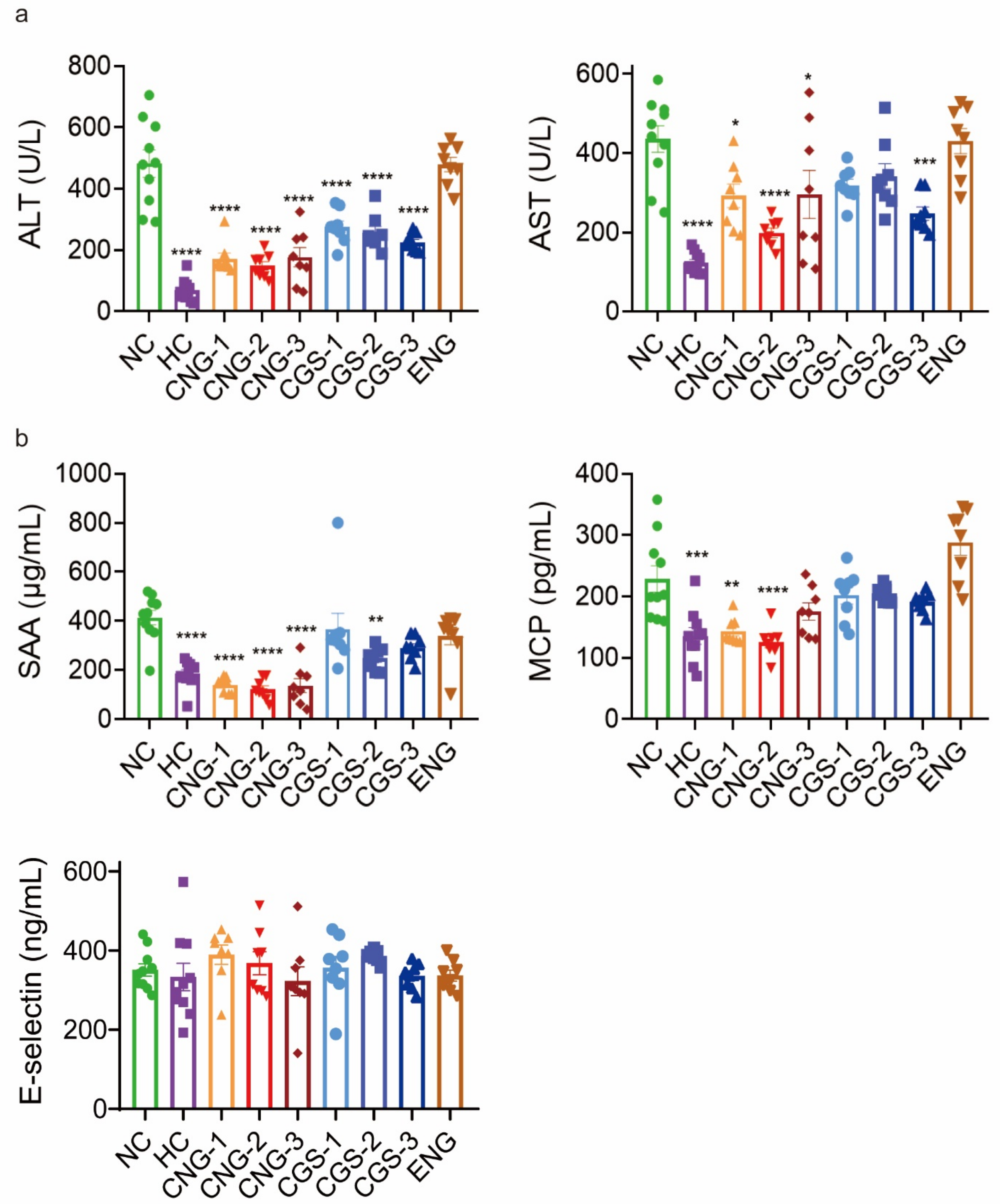

Figure S13. Effects of vehicle, CGS 26214 of three doses (CGS-1,2,3), CGS-ANG of three doses (CNG-1,2,3) and ENG on serum ALT, AST **(a)** and proinflammatory cytokines SAA, MCP, E-selectin **(b)** of mice in five weeks in therapeutic study. All data are shown as mean ± s.e.m. n= 8-10 biologically independent mice per group. Statistical significance was calculated via Ordinary one-way ANOVA with Tukey's multiple comparison test. *$P< 0.05$; **$P< 0.01$; ***$P< 0.001$; ****$P< 0.0001$ compared to NC. control.

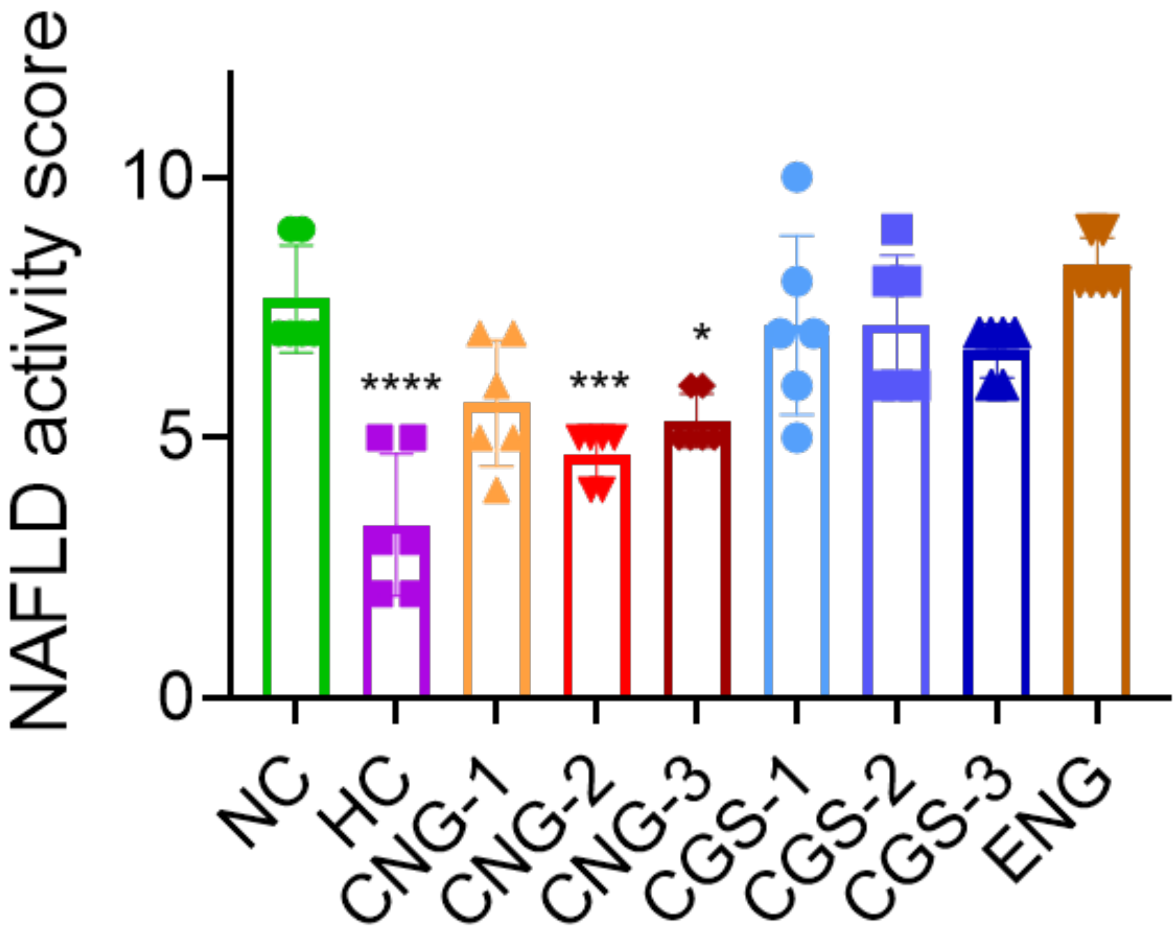

Figure S14. NAFLD activity scores of mice after treatments of vehicle, CGS 26214 of three doses (CGS-1,2,3), CGS-ANG of three doses (CNG-1,2,3) and ENG in therapeutic study. Cases with NAFLD activity score ≥ 5 were classified as NASH. n= 6 biologically independent mice per group. All data are shown as mean ± s.e.m. Statistical significance was calculated via Ordinary one-way ANOVA with Tukey's multiple comparison test. *P< 0.05; **P< 0.01; ***P< 0.001; ****P< 0.0001 compared to NC control.

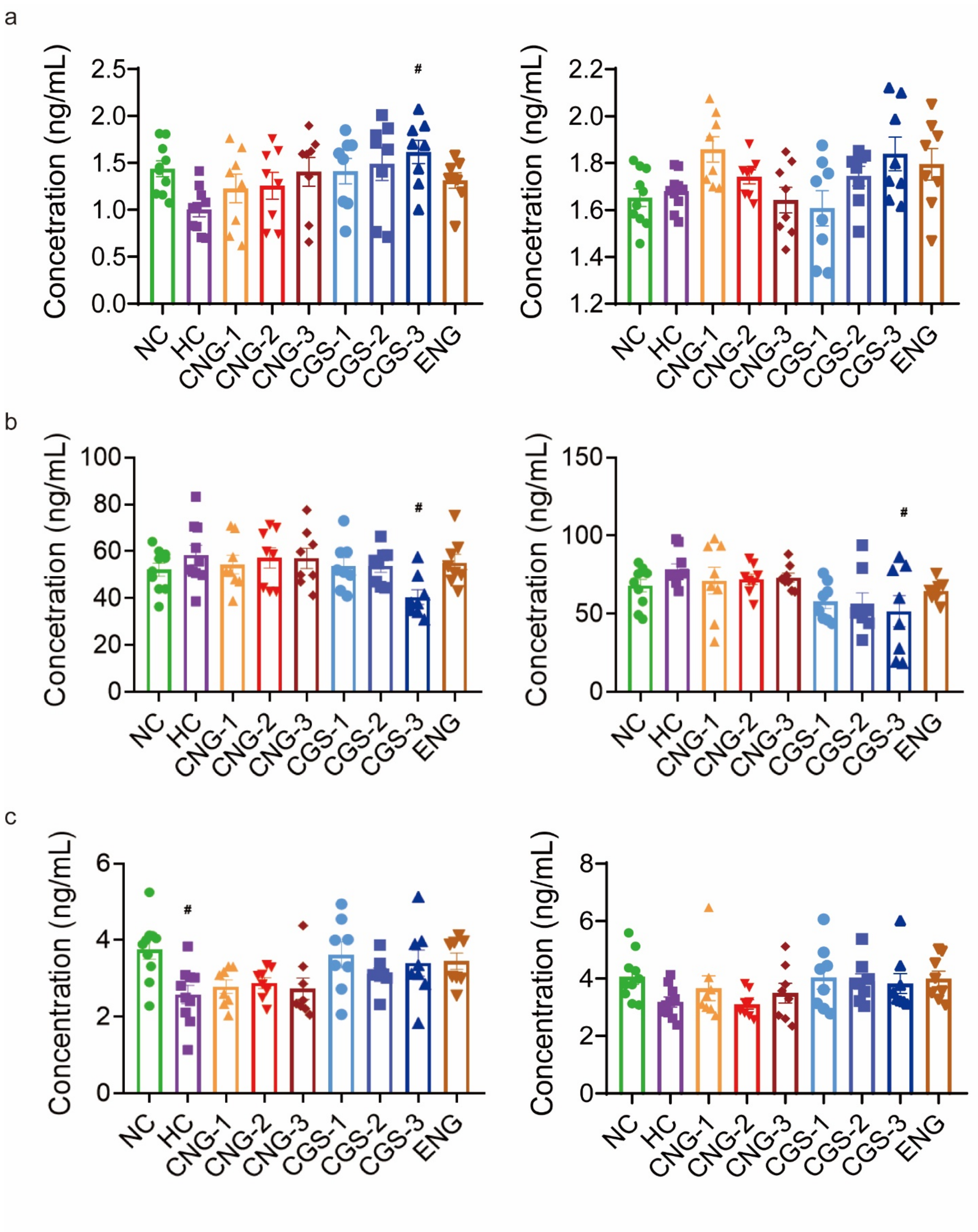

a
b
c
Concetration (ng/mL)
Concentration (ng/mL)
NC
HC
CNG-1
CNG-2
CNG-3
CGS-1
CGS-2
CGS-3
ENG
#

Figure S15. Effects of vehicle, CGS 26214 of three doses (CGS-1,2,3), CGS-ANG of three doses (CNG-1,2,3) and ENG on serum T3 **(a)**, T4 **(b)** and TSH **(c)** of mice in five weeks in preventing study (left) and therapeutic study (right). All data are shown as mean ± s.e.m. n= 8-10 biologically independent mice per group. Statistical significance was calculated via Ordinary one-way ANOVA with Tukey's multiple comparison test. $^{\#}P < 0.05$ compared to HC control.

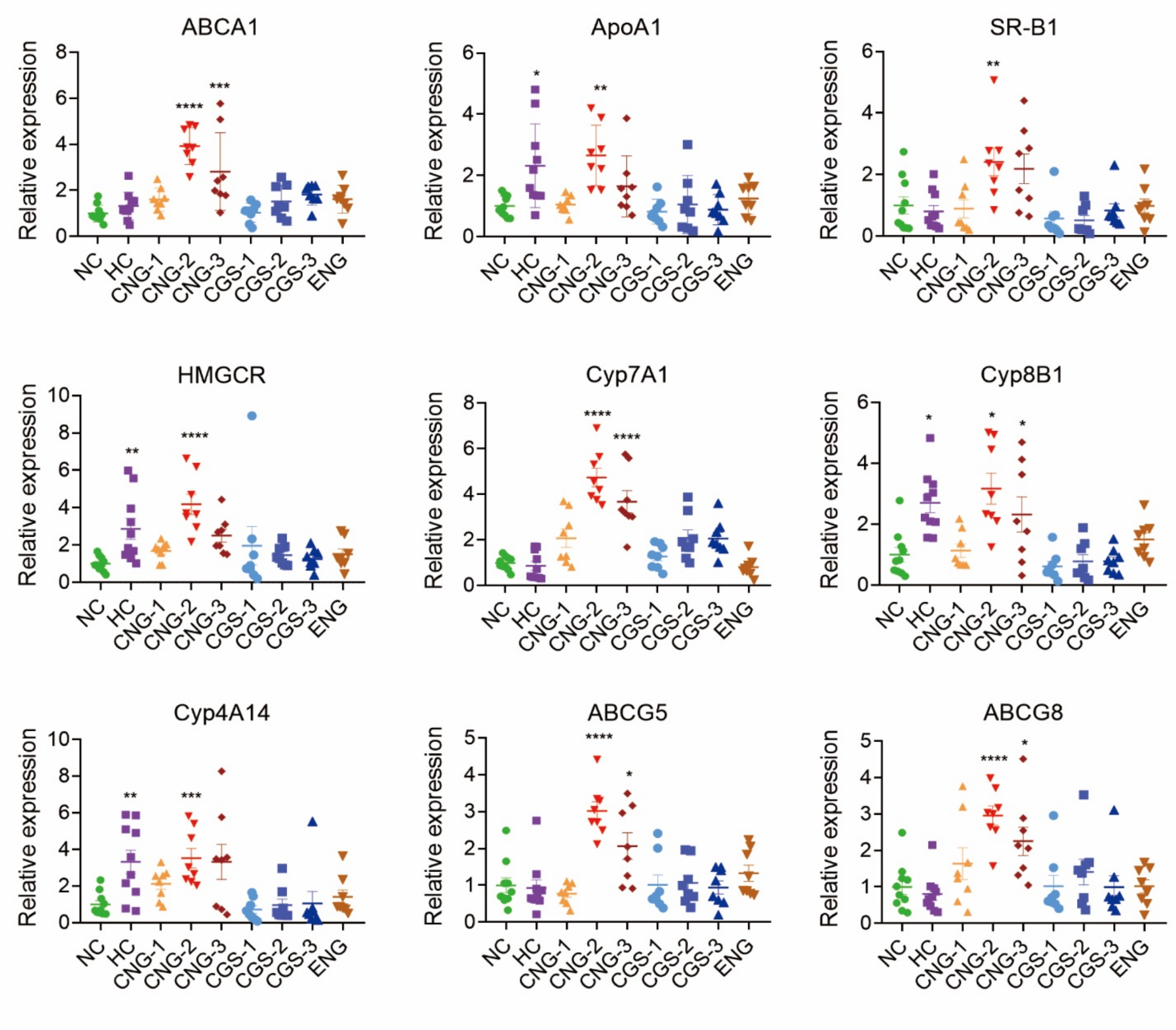

Figure S16. Relative mRNA expression of genes related to reverse cholesterol transport (RCT) in livers of mice after treatments of vehicle, CGS 26214 of three doses (CGS-1,2,3), CGS-ANG of three doses (CNG-1,2,3) and ENG in therapeutic study. n= 8-10 biologically independent mice per group. All data are shown as mean ± s.e.m. Statistical significance was calculated via Ordinary one-way ANOVA with Tukey's multiple comparison test. *P< 0.05; **P< 0.01; ***P< 0.001; ****P< 0.0001 compared to NC control.

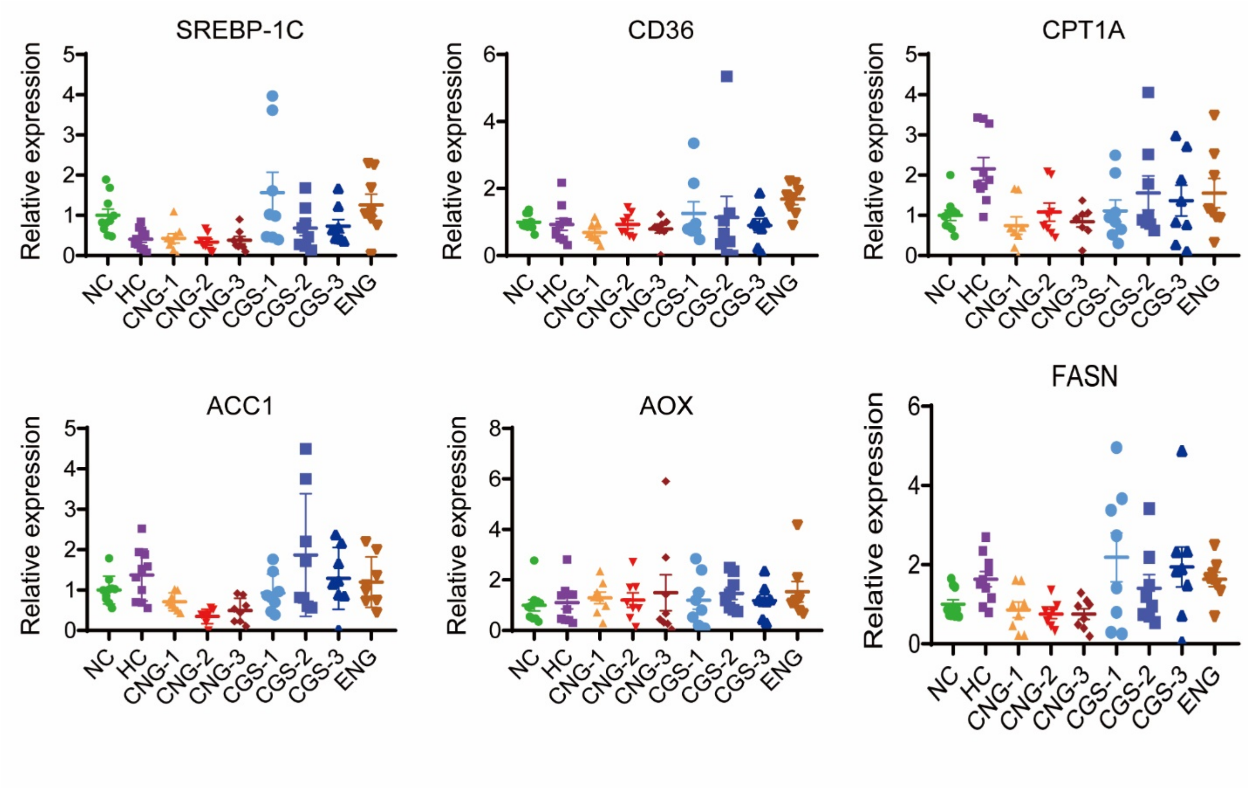

Figure S17. Relative mRNA expression of genes related to lipogenesis and fatty acid metabolism in livers of mice after treatments of vehicle, CGS 26214 of three doses (CGS-1,2,3), CGS-ANG of three doses (CNG-1,2,3) and ENG in therapeutic study. n= 8-10 biologically independent mice per group. All data are shown as mean ± s.e.m. Statistical significance was calculated via Ordinary one-way ANOVA with Tukey's multiple comparison test. *P< 0.05; **P< 0.01; ***P< 0.001; ****P< 0.0001 compared to NC control.

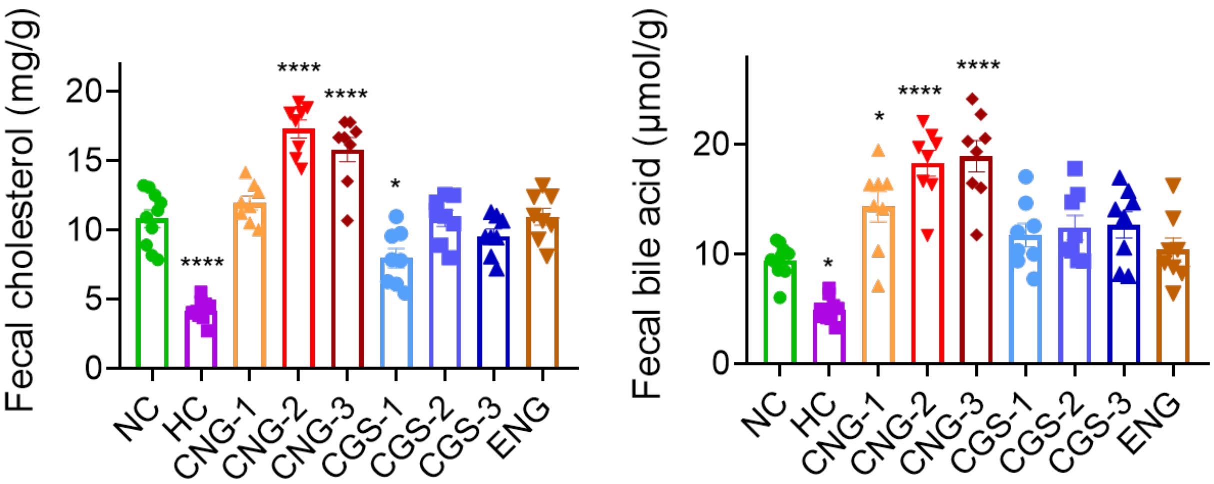

Figure S18. The levels of fecal cholesterol and fecal bile acid of mice after treatments of vehicle, CGS 26214 of three doses (CGS-1,2,3), CGS-ANG of three doses (CNG-1,2,3) and ENG in therapeutic study. n= 8-10 biologically independent mice per group. All data are shown as mean ± s.e.m. Statistical significance was calculated via Ordinary one-way ANOVA with Tukey's multiple comparison test. *P< 0.05; **P< 0.01; ***P< 0.001; ****P< 0.0001 compared to NC control.

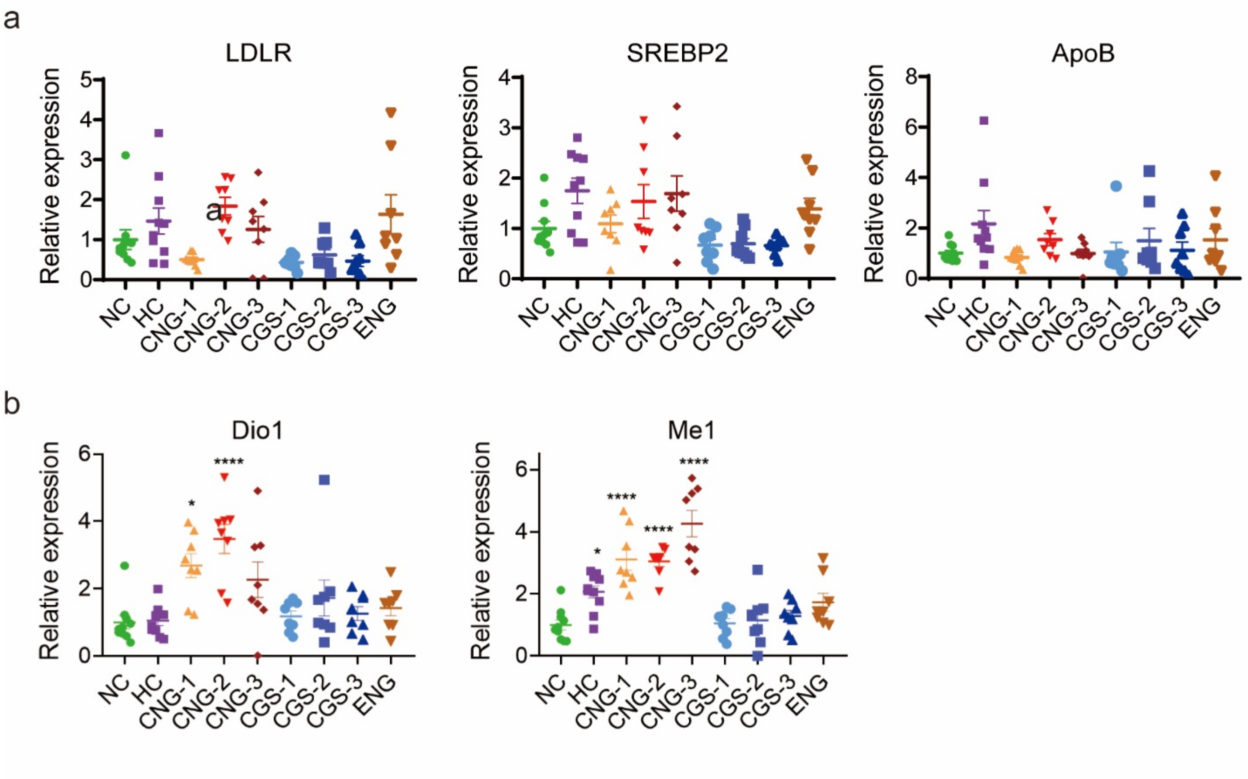

Figure S19. Relative mRNA expression of genes related to LDLR-mediated cholesterol transport **(a)** and THR activation **(b)** in livers of mice after treatments of vehicle, CGS 26214 of three doses (CGS-1,2,3), CGS-ANG of three doses (CNG-1,2,3) and ENG in therapeutic study. n= 8-10 biologically independent mice per group. All data are shown as mean ± s.e.m. Statistical significance was calculated via Ordinary one-way ANOVA with Tukey's multiple comparison test. *P< 0.05; **P< 0.01; ***P< 0.001; ****P< 0.0001 compared to NC control.

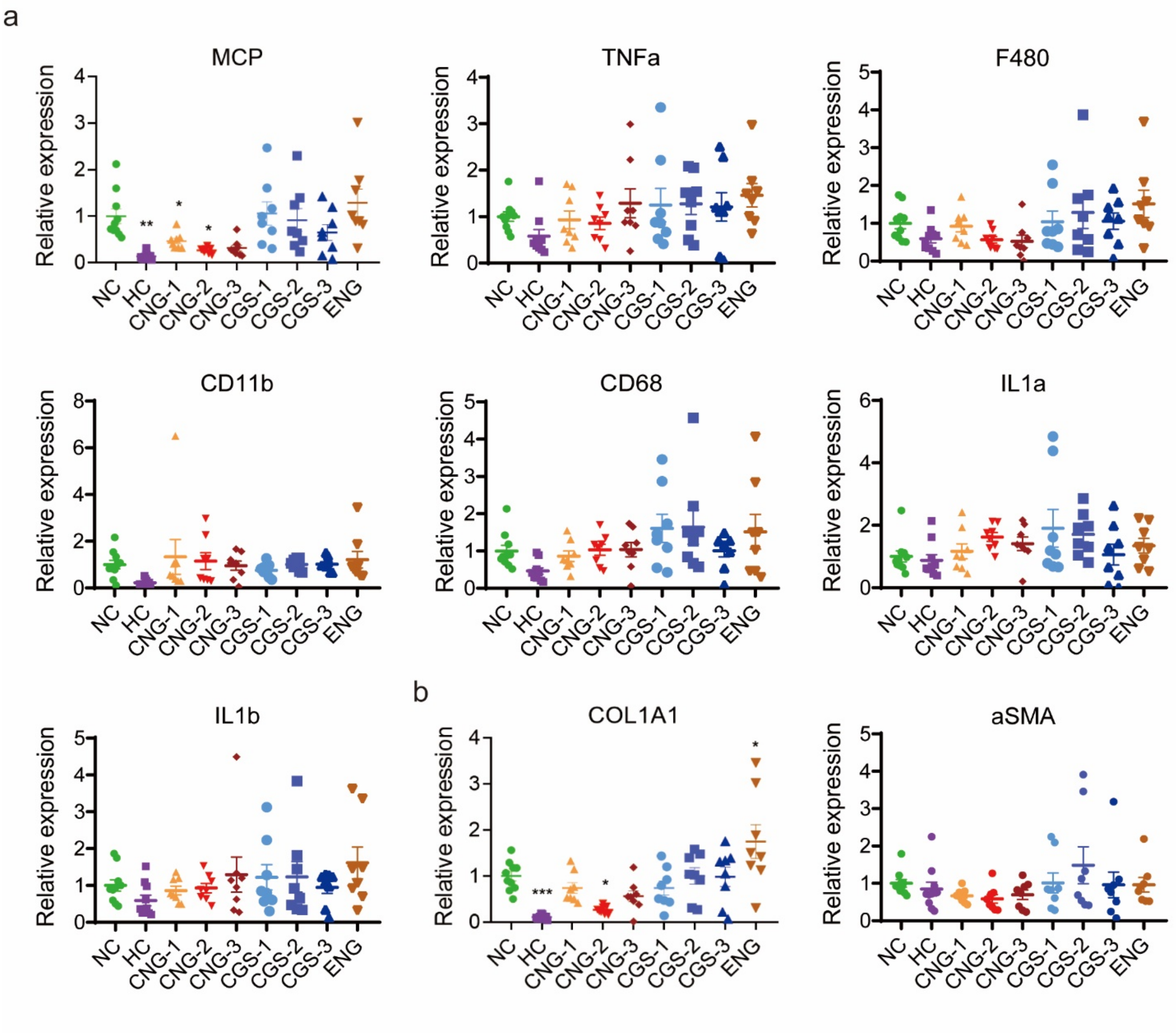

Figure S20. Relative mRNA expression of genes related to hepatic inflammation **(a)** and fibrosis **(b)** in livers of mice after treatments of vehicle, CGS 26214 of three doses (CGS-1,2,3), CGS-ANG of three doses (CNG-1,2,3) and ENG in therapeutic study. n= 8-10 biologically independent mice per group. All data are shown as mean ± s.e.m. Statistical significance was calculated via one-way ANOVA with Dunnett's test comparing each group to NC control. *P< 0.05; **P< 0.01; ***P< 0.001; ****P< 0.0001 compared to NC control.

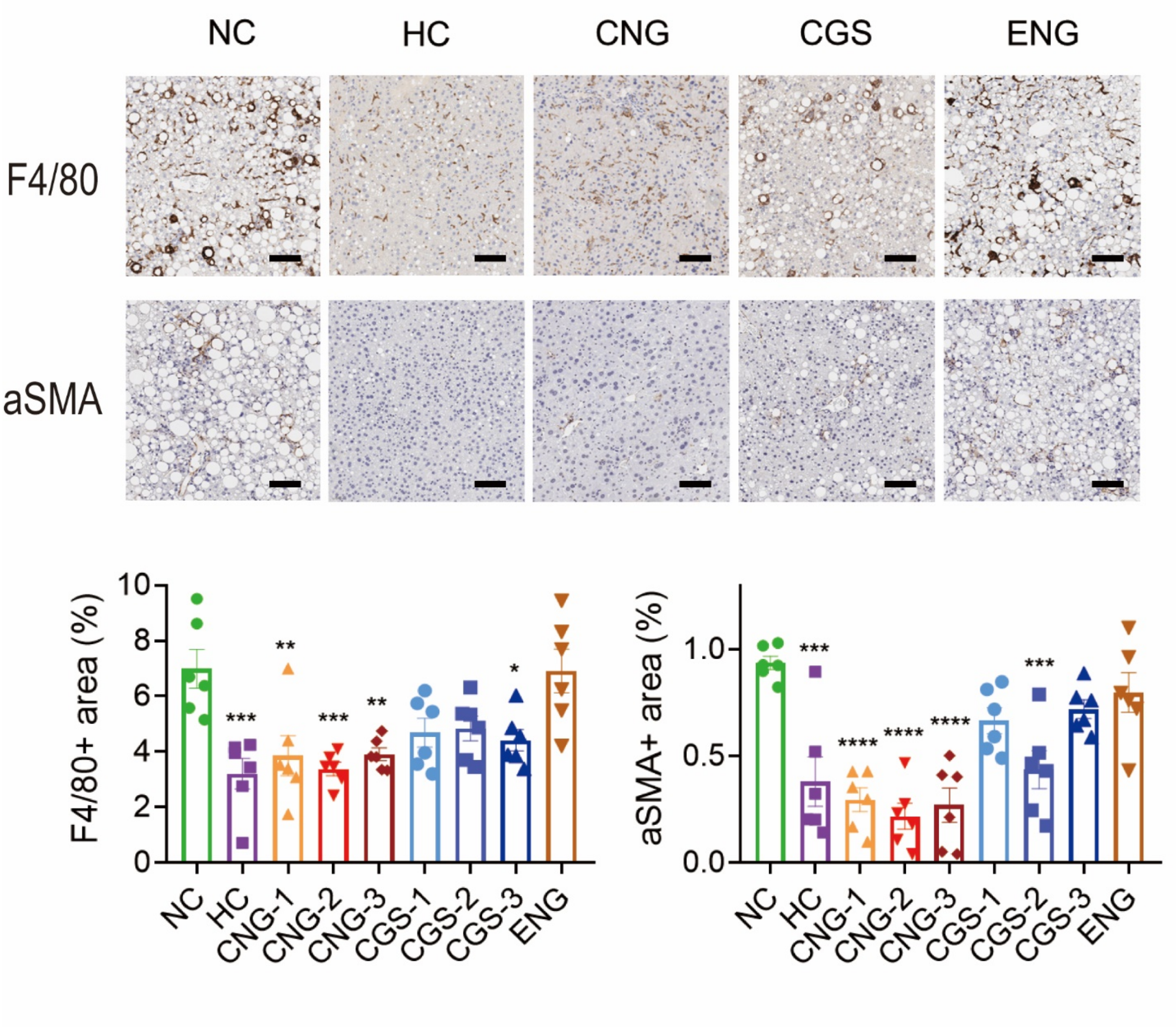

Figure S21. Representative immunohistochemistry images and quantitative analysis of liver F4/80 and α-SMA in therapeutic study. Scale bar, 100 µm. Positive areas were quantified from three randomly chosen fields per liver section from individual mice using six mice per group**.** CGS-2 and CNG-2 were chosen as the representative in CGS and CGS-ANG groups. n= 6 biologically independent mice per group. Statistical significance was calculated via Ordinary one-way ANOVA with Tukey's multiple comparison test. *P< 0.05; **P< 0.01; ***P< 0.001; ****P< 0.0001 compared to NC control.

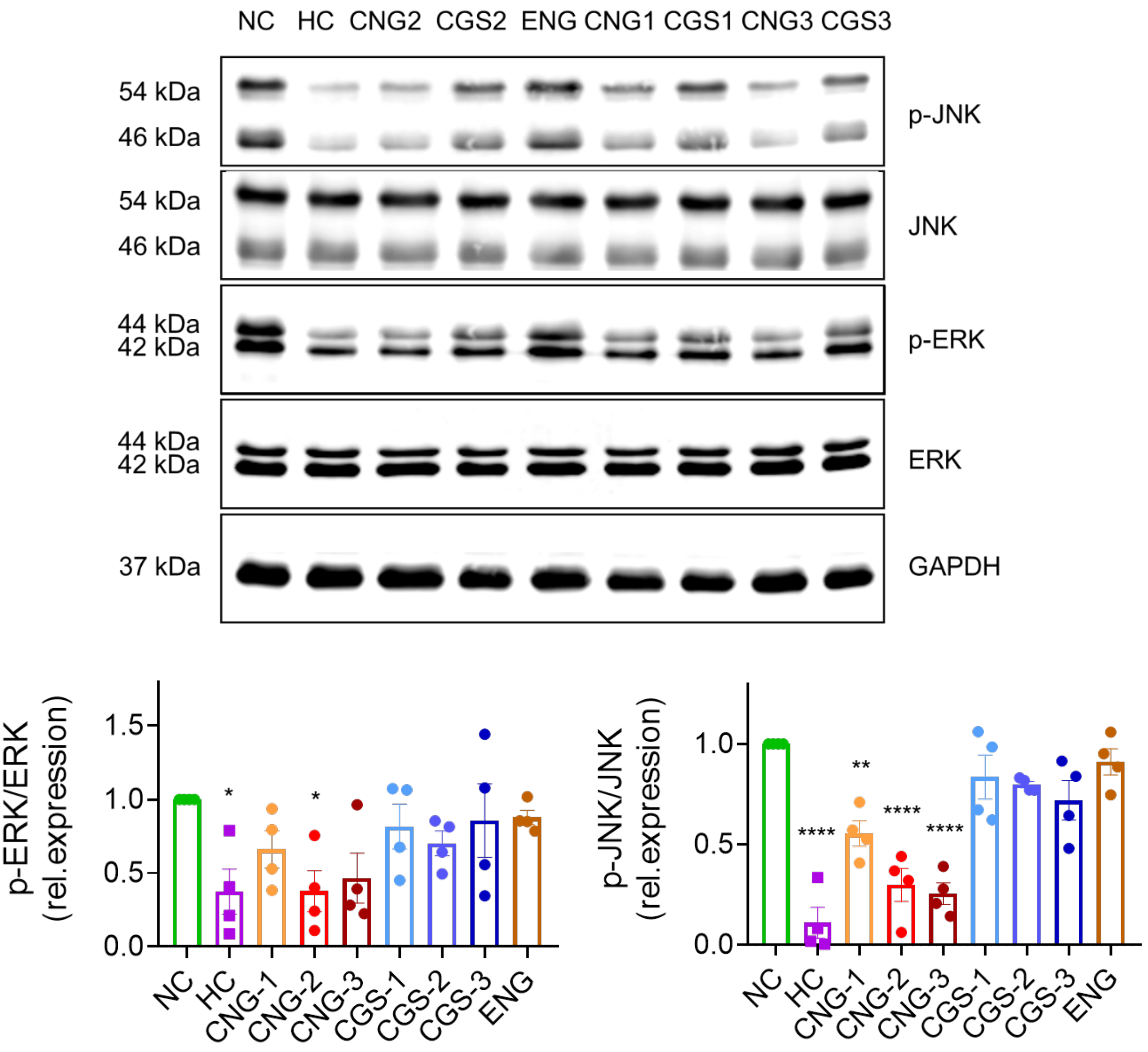

Figure S22. The protein levels of phospho-ERK1/2, ERK1/2, phospho-JNK, and JNK in the liver. The protein levels of phospho-ERK, ERK, phospho-JNK, and JNK in the livers of different treatment groups. The band densities for phospho-ERK, ERK, phospho-JNK, and JNK were first divided by GAPDH density to correct for very small loading differences. Then the levels of phospho-JNK/JNK and phospho-ERK/ERK were normalized to NC group. n= 8 biologically independent mice per group and every two mice were pooled as one sample. Cumulative densitometric analyses were performed from four independent gels. All data are shown as mean ± s.e.m. Statistical significance was calculated via Ordinary one-way ANOVA with Tukey's multiple comparison test. *$P < 0.05$; **$P < 0.01$; ***$P < 0.001$; ****$P < 0.0001$ compared to NC control.

Abbreviations: α-SMA, alpha-smooth muscle actin; ABCA1, ATP-binding cassette A1; ABCG5, ATP-binding cassette G5; ABCG8, ATP-binding cassette G8; ACC, acetyl CoA carboxylase; ADC, antibody-drug conjugate; ALT, alanine transaminase; ANG, anionic nanogel; AOX, alternative oxidase; ApoA1, apolipoprotein A1; AST, aspartate aminotransferase; CGS-1, CGS 26214 with low dose; CGS-2, CGS 26214 with medium dose; CGS-3, CGS 26214 with high dose; CGS-ANG, CGS 26214-encapsulated ANG; CNG-1, CGS-ANG with low dose; CNG-2, CGS-ANG with medium dose; CNG-3, CGS-ANG with high dose; COL1A1, collagen type I alpha 1; CPT1a, carnitine palmitoyltransferase 1A; Cy3-ANG, Cy3-labeled anionic nanogels; Cy3-NG, Cy3-labeled nanogels; Cy3-NNG, Cy3-labeled neutral nanogels; Cy7-ANG, Cy7-labeled anionic nanogels; Cy7-NNG, Cy7-labeled neutral nanogels; Cyp7A1, cholesterol 7 alpha-hydroxylase; Cyp8B1, sterol 12-alpha hydroxylase; DTT, dithiothreitol; DIO-NASH, diet-induced obesity-NASH; Dio1, Deiodinase 1; DLS, dynamic light scattering; ECM, extracellular matrix; EFP, Epididymal fat pads; ENG, empty nanogels; ERK, extracellular signal-regulated kinase; eWAT, epididymal white adipose tissue; FASN, fatty acid synthase; FGF15/19, fibroblast growth factor 15/19; GAN, gubra amylin NASH; GAPDH, glyceraldehyde 3-phosphate dehydrogenase; GPC, gel permeation chromatography; GSH, glutathione; H&E, hematoxylin and eosin; HC, healthy control; HDL, high density lipoproteins; HMGCR, 3-hydroxy-3-methyl-glutaryl CoA reductase; HSC, hematopoietic stem cells; JNK, Jun N-terminal kinase; LDL, low-density lipoprotein; LDLR, low-density lipoprotein receptor; MAPK, mitogen-activated protein kinase; MCP-1, monocyte chemoattractant protein-1; ME1, malic enzyme 1; NAFLD, non-alcoholic fatty liver disease; NAS, NAFLD activity score; NASH, non-alcoholic steatohepatitis; NC, NASH control; NMR, nuclear magnetic resonance; NNG, neutral nanogel; OATP, organic anion transporting polypeptides; PDSEMA, pyridyl disulfide ethyl methacrylate; RCT, reverse cholesterol transport; RXR, retinoid X receptor; SAA, serum amyloid A; SR-B1, scavenger receptor class B member 1; SREBP-1c, sterol regulatory element-binding protein-1c; SREBP-2, sterol regulatory element-binding protein-2; T4, thyroxine; TEM, transmission electron microscopy; TH, thyroid hormone; TREs, TH response elements; TSH, thyroid-stimulating hormone; VLDL, very-low-density lipoprotein
